# Supplementary material for: Delivery of Active Peptides by Self-Healing, Biocompatible and Supramolecular Hydrogels
Source: Molecules. 2023 Mar 10;28(6):2528. doi: 10.3390/molecules28062528 (PMC10057174; doi:10.3390/molecules28062528)
Supplement: Supplementary file 1 [file molecules-28-02528-s001.zip › molecules-2266403-supplementary.pdf]

SUPPORTING INFORMATION

Delivery of Active Peptides by Self-Healing, Biocompatible and Supramolecular Hydrogels

Seyedeh Rojin Shariati Pour,[a‡] Sara Oddis,[a‡] Marianna Barbalinardo,[b] Paolo Ravarino,[a] Massimiliano Cavallini,[b] Jessica Fiori,[a] Demetra Giuri,*[a] and Claudia Tomasini*[a]*

[a] *Diepartment of Chemistry Giacomo Ciamician, University of Bologna, Via Selmi, 2, 40126 Bologna, Italy*
[b] *National Research Council, Institute for the Study of Nanostructured Materials (CNR-ISMN), Via P. Gobetti 101, Bologna, 40129, Italy*

***** Correspondence: email: claudia.tomasini@unibo.it; jessica.fiori@unibo.it; demetra.giuri2@unibo.it

	Page
**Scheme S1.** Scheme of Synthesis of Compound **3**	S2
**Scheme S2.** Scheme of Synthesis of Compound **4**	S2
Synthesis and Characterization of Compounds **3** and **4**	S5–S7
^{1}H NMR, ^{13}C NMR, IR, HPLC-MS spectra of the described compounds	S8–S30

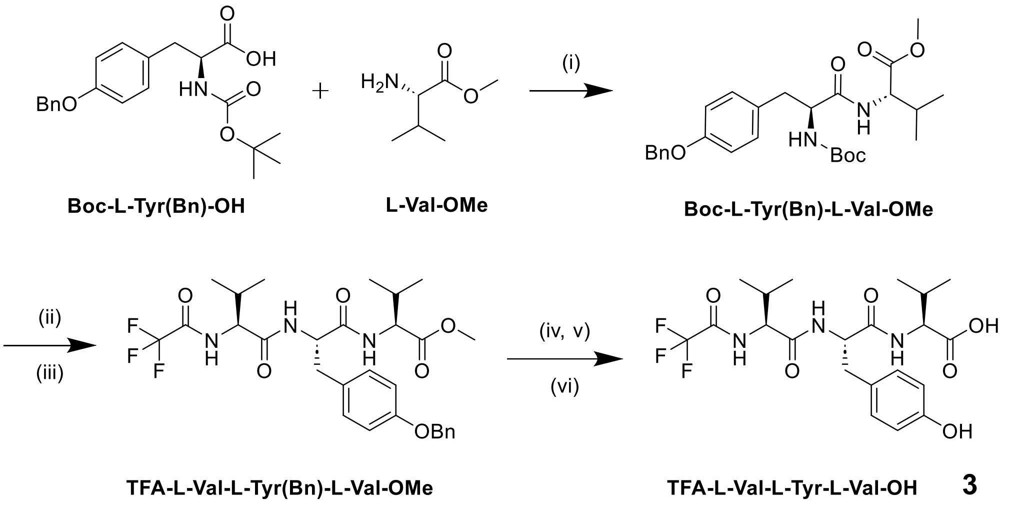

3

Scheme S1. Reagents and Conditions: (i) HBTU, DIEA, dry ACN, 2 h, r.t; (ii) TFA, dry CH_2Cl_2, 4 h, r.t.; (iii) TFA-L-Val-OH, HBTU, DIEA, dry ACN, 3 h, r.t.; (iv) MeOH, THF, NaOH, 18 h, r.t.; (v) HCl; (vi) 10% Pd/C, MeOH, 24 h, r.t.

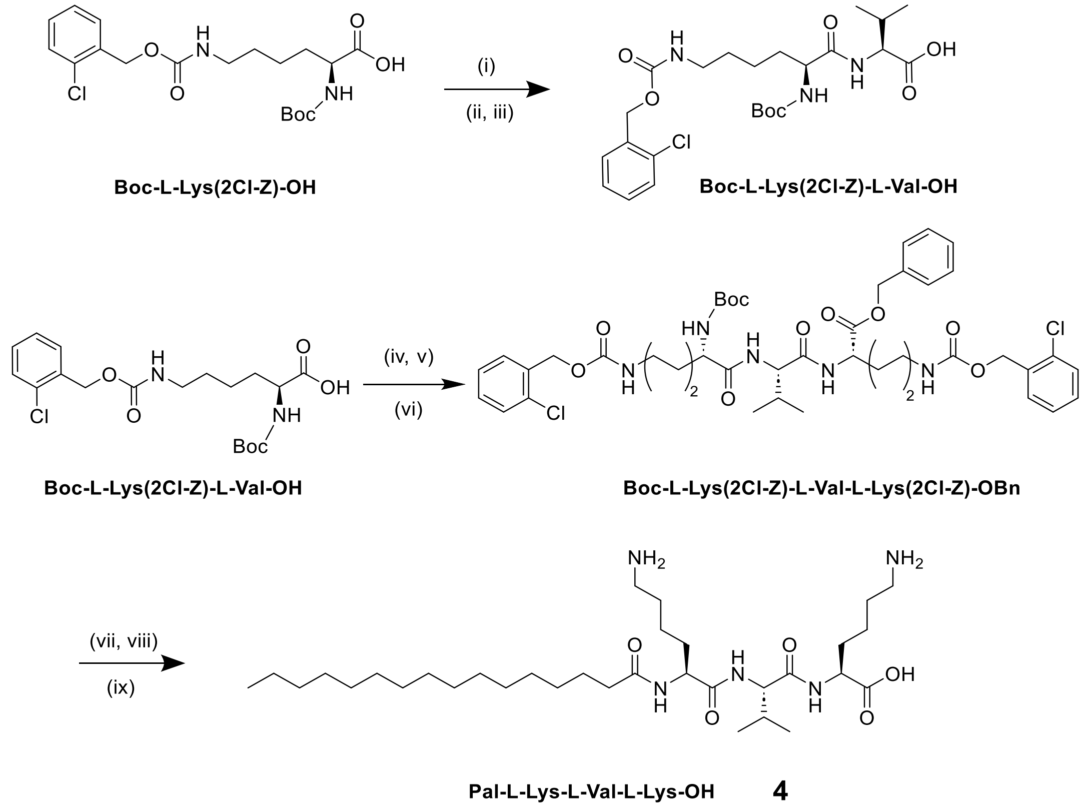

4

Scheme S2. Reagents and Conditions: (i) L-Val-OMe, HBTU, DIEA, dry ACN, 2 h, r.t.; (ii) MeOH, THF, NaOH, 18 h, r.t.; (iii) HCl; (iv) K_2CO_3, BnBr, dry ACN, 18 h, r.t.; (v) TFA, dry CH_2Cl_2, 2h, r.t.; (vi) Boc-L-Lys(2Cl-Z)-L-Val-OH, HBTU, DIEA, dry ACN, 3 h, r.t.; (vii) TFA, dry CH_2Cl_2, 4 h, r.t.; (vii) Palmitic acid, HBTU, DIEA, dry ACN, 4 h, r.t; (ix) 10% Pd/C, MeOH, 24 h, r.t.

Materials - All reactions were carried out in dried glassware and using dry solvents. The melting points of the compounds were determined in open capillaries and are uncorrected. High-quality infrared spectra (64 scans) were obtained at 2 cm^{-1} resolution with an ATR-IR Bruker (Billerica, US, MA) Alpha System spectrometer. All compounds were dried in vacuo and all the sample preparations were performed in a nitrogen atmosphere. NMR spectra were recorded with a Varian (Palo Alto, US, CA) Inova 400 spectrometer at 400 MHz (^{1}H NMR) and at 100 MHz (^{13}C NMR). Chemical shifts are reported in δ values relative to the solvent peak. HPLC-MS analysis was carried out with an Agilent 1260 Infinity II liquid chromatography coupled to an electrospray ionization mass spectrometer (LC-ESI-MS), using a Phenomenex Gemini C18 - 3μ - 110 Å column, H_2O/CH_3CN with 0.2% formic acid as acid solvent at 40 °C (positive ion mode, mz = 50-2000, fragmentor 70 V).

Boc-L-Tyr(Bn)-L-Val-OMe - Boc-L-Tyr(Bn)-OH (500 mg, 1.346 mmol) and HBTU (561 mg, 1.481 mmol) were dissolved in 15 mL of dry ACN. L-Valine-OMe.HCl (226 mg, 1.346 mmol), DIEA (0.733 ml, 4.307 mmol) and 10 mL of dry ACN were added dropwise to the mixture. The reaction solution was stirred at room temperature for 2 h under a N_2 atmosphere. The solvent was evaporated and the residue was dissolved in EtOAc. The residue was washed with HCl 1M (x2), $NaHCO_3$ (x2) and Brine (x1). The organic phase was dried over Na_2SO_4. The solvent was evaporated and Boc-L-Tyr(Bn)-L-Val-OMe was obtained in 95% yield (620 mg, 1.279 mmol). HPLC-MS (ESI): 9.64 min, $[(M\text{-}Boc)+H]^+$ = 385, $[M+Na]^+$ = 507. ^{1}HNMR ($CDCl_3$, 400 MHz): δ 0.84 (dd, 6H, *J* = 6.8, 11.6 Hz, $(CH_3)_2$ Val), 1.40 (s, 9H, Boc), 2.08 (m, 1H, C*H*$(CH_3)_2$ Val), 2.99 (d, 2H, *J* = 6.4 Hz, CH_2 Tyr), 3.67 (s, 3H, O-C*H₃*), 4.30 (bs, 1H, $C_\alpha H$ Tyr), 4.44 (dd, 1H, *J* = 5.2, 8.4 Hz, $C_\alpha H$ Val), 5.01 (s, 2H, O-CH_2Ph Tyr), 5.04 (bs, 1H, N*H*Boc), 6.40 (d, 1H, J = 8.4 Hz, N*H* Val), 6.87 (d, 2H, J = 8.4 Hz, Ph Tyr), 7.10 (d, 2H, J = 8.3 Hz, Ph Tyr), 7.26-7.40 (m, 5H, Bn). ^{13}CNMR ($CDCl_3$, 100 MHz): δ 17.76, 18.79, 21.58, 28.24, 31.25, 37.14, 52.04, 55.91, 57.22, 69.96, 80.16, 114.97, 127.39, 127.91, 128.54, 128.82, 130.34, 136.96, 155.40, 157.78, 171.22, 171.76.

TFA-L-Val-L-Tyr(Bn)-L-Val-OMe - Boc-L-Tyr(Bn)-L-Val-OMe (500 mg, 1.032 mmol) and TFA (1.43 mL, 18.57 mmol) were added to dry CH_2Cl_2 (6 mL). The reaction was stirred at room temperature for 2 h under a N_2 atmosphere, then the solvent was removed under reduced pressure. The product was obtained in quantitative yield and used for the following reaction without any further purification.

TFA-L-Val-OH was obtained as previously reported.[1] Trifluoroacetic anhydride (0.840 mL, 6.074 mmol) was added to a solution of L-Valine (593 mg, 5.062 mmol) in trifluoroacetic acid (2.5 mL) and the resulting mixture was stirred for 1.5 h. TFA was then evaporated under reduced pressure. Water was added and the residue was extracted with EtOAc (3x). The organic layer was then dried over Na_2SO_4 and the solvent was evaporated. The product was washed with n-hexane and obtained in 96% yield (1.035 g, 4.860 mmol). The characterization matched the values reported in reference.

TFA-L-Val-OH (220 mg, 1.032 mmol) and HBTU (430 mg, 1.135 mmol) were dissolved in 10 ml of dry ACN. $NH_3^+TFA^-$-L-Tyr(Bn)-L-Val-OMe (398 mg, 1.032 mmol) and DIEA (1.36 mL, 8.0 mmol) were dissolved in dry ACN (5 mL) and added dropwise to the mixture. The reaction solution was stirred at room temperature for 3 h under N_2 atmosphere. The solvent was removed and the residue was dissolved in EtOAc and was washed with HCl 1M (x2), $NaHCO_3$ (x2) and Brine (x1). The organic layer was then dried over Na_2SO_4. The solvent was evaporated under reduced pressure. The residue was washed with n-hexane (2x) and the product obtained in an 82% yield (504 mg, 0.846 mmol). HPLC-MS (ESI): 9.09 min, $[M+H]^+$ = 580, $[M+Na]^+$ = 602. ^{1}HNMR (CD_3OD, 400 MHz, mixture of conformers): δ 0.57 – 0.76 (3H, m, $(CH_3)_2$ Val), 0.90 (9H, m, 3 CH_3 Val), 1.86 (1H, m, C*H*$(CH_3)_2$ Val), 2.03 (1H, m, C*H*$(CH_3)_2$ Val), 2.84 – 3.01 (m, 2H, C*H*$_2$ Tyr), 3.65 (s, 3H, OMe), 4.09+4.18 (1H, m, C_α*H* Val), 4.28 (1H, m, C_α*H* Val), 4.65 (1H, m, C_α*H* Tyr), 5.03 (bs, 2H, C*H*$_2$Ph Tyr), 6.87 – 7.14 (m, 4H, Ph Tyr), 7.30 – 7.40 (m, 5H, Bn Tyr). ^{13}C NMR (CD_3OD, 100 MHz): δ 17.03, 17.46, 17.96, 18.11, 29.96, 30.28, 30.54, 36.74, 51.01, 54.54, 57.75, 59.35, 69.48, 114.35, 114.56, 127.04, 127.35, 128.02, 128.82, 129.95, 137.39, 157.69, 170.44, 171.62, 171.82. ^{19}F NMR (CD_3OD, 376.5 MHz): δ -76.82.

TFA-L-Val-L-Tyr-L-Val-OH (3) - TFA-L-Val-L-Tyr(Bn)-L-Val-OMe (246 mg, 0.414 mmol) was dissolved in 0.66 ml of MeOH and 1.32 ml of THF. The reaction mixture was placed in an ice bath before the addition of NaOH 1M (0.518 mL, 0.518 mmol). The mixture was left under stirring overnight. After 18 h, 1M HCl (0.580 ml, 0.580 mmol) was added. The solvents were removed under reduced pressure and the residue was dissolved in DCM and washed with water. The organic layer was dried over Na_2SO_4 and the solvent evaporated. The product was obtained in 95% yield (229 mg, 0.393 mmol).

TFA-L-Val-L-Tyr(Bn)-L-Val-OH (230 mg, 0.396 mmol) was dissolved in 23 mL of methanol (10% V/w) in presence of Pd/C (10% w/w) and a H_2 atmosphere. The reaction was left under stirring for 24 h then the solution was filtered through a celite pad and the solvent removed under reduced pressure. The product TFA- L-Val-L-Tyr-L-Val-OH was obtained as a white solid in 99% yield (193

mg, 0.392 mmol). $[\alpha]_D^{20}$ = -14.6 (*c* = 5 mg/mL, MeOH). m.p.: 150 - 152 °C. HPLC-MS (ESI): 4.29 min, $[M+H]^+$ = 476, $[M+Na]^+$ = 498. ^{1}HNMR (CD_3OD, 400 MHz, mixture of conformers): δ 0.61 – 0.91 (m, 12H, 2 $(CH_3)_2$ Val), 1.89 – 2.12 (m, 2H, 2 C*H*$(CH_3)_2$ Val), 2.76 - 3.07 (2H, m, C*H*$_2$Ph-Tyr), 4.14 (m, 1H, $C_\alpha H$ Val), 4.23 – 4.33 (m, 1H, $C_\alpha H$ TFA-Val), 4.73 (m, 1H, $C_\alpha H$ Tyr), 6.65 (m, 2H, Ph Tyr), 7.00 (m, 2H, Ph Tyr), 7.97-8.10 (m, 1H, NH TFA-Val), 8.40 (m, 1H, NH Tyr), 8.90 (m, 1H, NH Val). ^{13}CNMR (CD_3OD, 100 MHz): δ 18.21, 18.33, 18.60, 18.86, 19.38, 19.51, 31.72, 31.90, 37.48, 38.27, 55.96, 58.89, 60.72, 116.10, 128.76, 129.13, 131.30, 157.09, 157.19, 171.84, 173.38, 174.40. FTR-IR: ν 3277, 2963, 2412, 2163, 2011, 1973, 1702, 1643, 1549, 1514 cm^{-1}.

Boc-L-Lys(2Cl-Z)-L-Val-OH - Boc-L-Lys(2Cl-Z)-OH (400 mg, 0.964 mmol) and HBTU (379 mg, 1.05 mmol) were dissolved in dry ACN (10 mL). L-Valine-OMe.HCl (161 mg, 0.964 mmol), DIEA (0.525 mL, 3.085 mmol) and dry ACN (5 mL) were added dropwise to the mixture. The reaction solution was stirred at room temperature for 2 h under a N_2 atmosphere. The solvent was evaporated and the residue was dissolved in EtOAc. The residue was washed with HCl 1M (x2), $NaHCO_3$ (x2) and Brine (x1). The organic phase was dried over Na_2SO_4. The solvent was evaporated and Boc-L-Lys(2Cl-Z)-L-Val-OMe was obtained in 99% yield (471 mg, 0.954 mmol).

Boc-Lys(2Cl-Z)-Val-OMe (471 mg, 0.954 mmol) was dissolved in 1.88 ml of MeOH and 3.76 ml of THF. The reaction mixture was placed in an ice bath and NaOH 1M (1.2 mL, 1.20 mmol) was added. The mixture was left under stirring at r.t. overnight. After 18 h, 1M HCl (1.4 ml, 1.40 mmol) was added and after 10 min, the solvent was removed under reduced pressure. The residue was dissolved in DCM, washed with water (2x) and dried over Na_2SO_4. The solvent was evaporated and the product Boc-Lys(2Cl-Z)-Val-OH was obtained in 98% yield (480 mg, 0.935 mmol). HPLC-MS (ESI): 7.31 min, $[(M\text{-}Boc)+H]^+$ = 414, $[M+Na]^+$ = 536. ^{1}H NMR (400 MHz, $CDCl_3$): δ 0.93 (dd, 6H, J = 6.8, 10.4 Hz, $(CH_3)_2$ Val), 1.39 (s, 9H, Boc), 1.30-1.80 (m, 6H, *CH$_2$*-Lys), 2.21 (m, 1H, C*H*$(CH_3)_2$ Val), 3.15 (m, 2H, *CH$_2$*NH Lys), 4.15 (m, 1H, $C_\alpha H$ Val), 4.50 (m, 1H, $C_\alpha H$ Lys), 5.19 (s, 2H, *CH$_2$*(2Cl-Z)), 6.24 (bs, 1H, NH Val), 7.21-7.38 (m, 4H, Lys(*2Cl-Z*)), 6.85 – 6.94 (bs, 2H, NH Lys). ^{13}CNMR (CD_3OD, 100 MHz): δ 17.71, 19.18, 22.60, 28.44, 29.51, 30.92, 31.93, 38.78, 40.74, 54.40, 57.34, 64.11, 64.76, 80.44, 127.00, 129.46, 129.61, 129.84, 133.65, 134.43, 156.16, 156.66, 172.76, 174.46.

Boc-L-Lys(2Cl-Z)-L-Val-L-Lys(2Cl-Z)-OBn - Boc-L-Lys(2Cl-Z)-OH (500 mg, 1.205 mmol), dried K_2CO_3 (250 mg, 1.807 mmol) and benzyl bromide (0.157 mL, 1.326 mmol) were dissolved in dry ACN (10 mL). The reaction was left under stirring at r.t overnight in an N_2 atmosphere. The

solvent was then removed under reduced pressure. The residue was extracted with EtOAc (3x) and dried over Na_2SO_4. The solvent was removed and the crude was purified with silica gel chromatography (4:1 to 3:1 cHex: EtOAc). The product Boc-L-Lys(2Cl-Z)-OBn was obtained in 90% yield (547 mg, 1.084 mmol). HPLC-MS (ESI): 10.14 min, $[(M\text{-}Boc)+H]^+$ = 405, $[M+Na]^+$ = 527.

Boc-L-Lys(2Cl-Z)-OBn (547 mg, 1.084 mmol) was dissolved in 8 ml of dry CH_2Cl_2. TFA (1.4 ml, 17.82 mmol) was added to the flask. The reaction was left under stirring in an N_2 atmosphere for 2 h. The solvent was removed and the residue ($TFA^-NH_3^+$-L-Lys(2Cl-Z)-OBn) was used in the next step without further purification.

Boc-L-Lys(2Cl-Z)-L-Val-OH (480 mg, 0.935 mmol) and HBTU (390 mg, 1.028 mmol) were dissolved in dry ACN (8 mL). $NH_3^+TFA^-$-L-Lys(2Cl-Z)-OBn (379 mg, 0.935 mmol) and DIEA (0.850 mL, 5.0 mmol) were dissolved in dry ACN (5 mL) and added dropwise to the mixture. The reaction solution was stirred at room temperature for 3 h under N_2 atmosphere. The solvent was removed and the residue was dissolved in CH_2Cl_2 and was washed with HCl 1M (x2), $NaHCO_3$ (x2) and Brine (x1). The organic layer was then dried over Na_2SO_4. The solvent was evaporated under reduced pressure. The residue was purified with silica chromatography (4:1 to 3:1 cHex:EtOAc) and the product Boc-L-Lys(2Cl-Z)-L-Val-L-Lys(2Cl-Z)-OBn was obtained in a 70% yield (504 mg, 0.846 mmol). HPLC-MS (ESI): 11.10 min, $[(M\text{-}Boc)+H]^+$ = 802, $[M+H_3O]^+$ = 920. ^{1}H NMR (400 MHz, $CDCl_3$): δ 0.89 (m, 6H, 2CH_3 Val), 1.30 – 1.50 (m, 4H, 2 CH_2 Lys), 1.38 (s, 9H, Boc), 1.55 – 1.80 (m, 6H, 2H C*H*$_2$CHNH Lys, 4H CH_2 Lys), 1.82 (m, 2H, C*H*$_2$CHNH Lys), 2.10 (m, 1H, C*H*NH Val), 3.03-3.20 (m, 4H, 2 C*H*$_2$NH Lys), 4.05 (m, 1H, $C_\alpha H$ Lys), 4.20 (m, 1H, $C_\alpha H$ Val), 4.56 (m, 1H, $C_\alpha H$ Lys), 5.05 – 5.24 (m, 6H, 2 *CH*$_2$(2Cl-Ph), *CH*$_2$ (Bn)), 5.25 – 5.42 (m, 3H, N*H*COO Lys), 6.75 (bs, 2H, NH Val, NH Lys), 7.17-7.38 (13H, O*Bn* Val, (2Cl-*Z*) Lys). ^{13}C NMR (100 MHz, $CDCl_3$): δ 17.94, 19.20, 21.44, 22.15, 22.35, 28.26, 29.07, 29.31, 30.37, 31.45, 40.26, 40.40, 51.98, 54.47, 58.82, 63.79, 67.14, 80.16, 126.79, 128.32, 128.45, 128.56, 129.20, 129.39, 129.60, 133.37, 134.33, 135.20, 155.83, 156.39, 170.89, 171.81, 172.42.

Pal-L-Lys-L-Val-L-Lys-OH - Boc-L-Lys(2Cl-Z)-L-Val-L-Lys(2Cl-Z)-OBn (504 mg, 0.846 mmol) was dissolved in 7 ml of dry CH_2Cl_2. TFA (1.2 ml, 15.55 mmol) was added to the flask. The reaction was left under stirring in an N_2 atmosphere for 4 h. The solvent was removed and $TFA^-NH_3^+$-L-Lys(2Cl-Z)-L-Val-L-Lys(2Cl-Z)-OBn was obtained in quantitative yield and used in the next step without further purification.

Palmitic acid (128 mg, 0.500 mmol) and HBTU (209 mg, 0.55 mmol) were dissolved in dry ACN (8 mL). $TFA^{-}NH_3^{+}$-L-Lys(2Cl-Z)-L-Val-L-Lys(2Cl-Z)-OBn (457 mg, 0.500 mmol) and DIEA (0.340 mL, 2.00 mmol) were dissolved in dry ACN (5 mL) and added dropwise to the mixture. The reaction solution was stirred at room temperature for 4 h under N_2 atmosphere. The solvent was removed and H_2O was added. The residue was extracted with CH_2Cl_2 (3x) and washed with HCl 1M (x2), $NaHCO_3$ (x2) and Brine (x1). The organic layer was then dried over Na_2SO_4. The solvent was evaporated under reduced pressure. The product Pal-L-Lys(2Cl-Z)-L-Val-L-Lys(2Cl-Z)-OBn was then washed with n-hexane (2x) and obtained in 85% yield (441 mg, 0.425 mmol). ^{1}H NMR (400 MHz, $CDCl_3$): δ 0.87 (m, 9H, $(CH_3)_2$ Val, CH_3 Pal), 1.07 – 1.90 (m, 34H, $(CH_2)_3$ 2 Lys, $(CH_2)_{12}CH_3$ Pal), 2.13 (m, 5H, $CH(CH_3)_2$ Val, $CO(CH_2)_2$ Pal), 3.11 (m, 4H, 2 CH_2NH Lys), 5.38 (m, 2H, NHCOO 2 Lys), 6.38 (bs, 1H, NH Lys), 7.19 – 7.35 (m, 13H, 5H Ph, 8H 2 2Cl-Z Lys). ^{13}C NMR (100 MHz, $CDCl_3$): δ 14.09, 18.01, 19.19, 22.17, 22.65, 25.67, 29.12, 29.33, 29.52, 29.66, 30.51, 31.51, 31.89, 36.38, 40.14, 40.43, 51.97, 52.96, 58.86, 63.79, 67.14, 74.84, 126.79, 128.29, 128.46, 128.56, 129.21, 129.39, 129.63, 133.40, 134.36, 135.17, 156.40, 170.92, 171.87, 173.68.

Pal-L-Lys(2Cl-Z)-L-Val-L-Lys(2Cl-Z)-OBn (441 mg, 0.425 mmol) was dissolved in MeOH (40 mL) (10% V/w) in presence of Pd/C (10% w/w) and a H_2 atmosphere. The reaction was left under stirring for 24 h, then the solution was filtered through a paper pad and the solvent removed under reduced pressure. The product Pal-L-Lys-L-Val-L-Lys-OH was obtained as a waxy solid in 84% yield (218 mg, 0.357 mmol). $[\alpha]_D^{20}$ = - 41.7 (*c* = 2.5 mg/mL, H_2O). HPLC-MS (ESI): 5.56 min, $[M-H]^-$ = 610. ^{1}H NMR (400 MHz, D_2O): δ 0.74 – 0.90 (m, 9H, $(CH_3)_2$ Val, CH_3 Pal), 1.14 – 1.50 (m, 28H, $(CH_2)_{12}$ Pal, 2 CH_2 Lys), 1.60 – 1.85 (m, 8H, 4 CH_2 Lys), 1.96 – 2.34 (m, 5H, $CH(CH_3)_2$ Val, 2 CH_2 Lys), 2.94 (m, 4H, 2 CH_2 Lys), 4.08 – 4.31 (m, 3H, $C_\alpha H$ Lys, $C_\alpha H$ Val, $C_\alpha H$ Lys). ^{13}C NMR (100 MHz, D_2O): δ 12.16, 13.88, 16.33, 17.79, 18.46, 20.80, 22.62, 25.46, 25.85, 26.40, 27.62, 29.46, 29.97, 31.95, 35.61, 39.27, 42.59, 44.39, 50.61, 53.04, 54.41, 173.36, 174.31, 174.95, 175.41. IR (ATR-IR): ν 3291, 3059, 2917, 2851, 1626, 1533 cm^{-1}.

Boc-L-Tyr(Bn)-L-Val-OMe (^{1}H NMR)

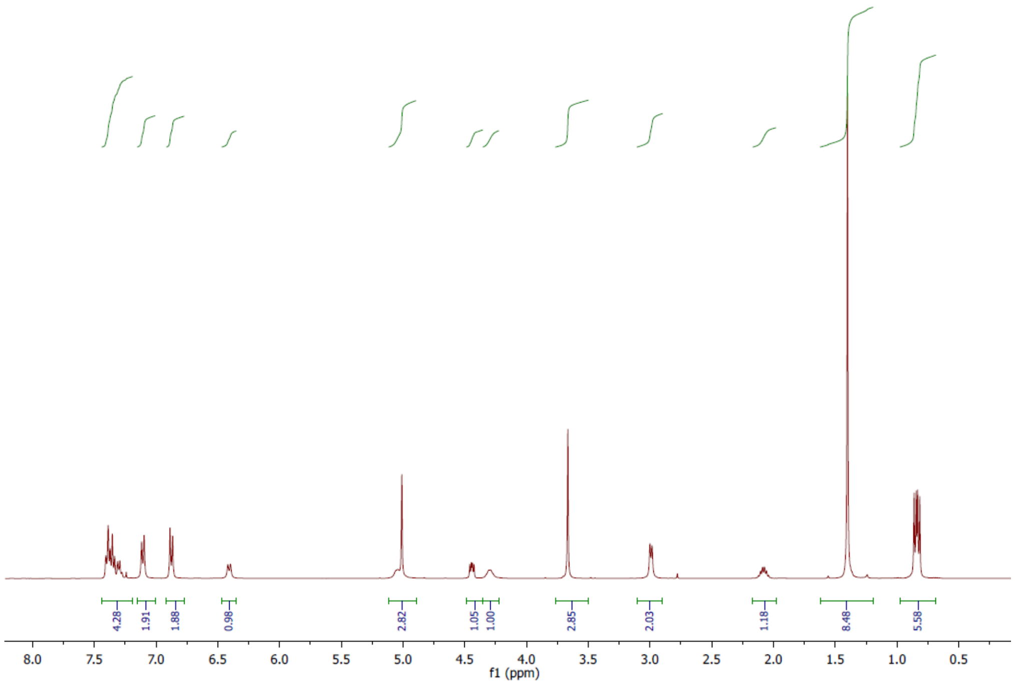

Boc-L-Tyr(Bn)-L-Val-OMe (COSY)

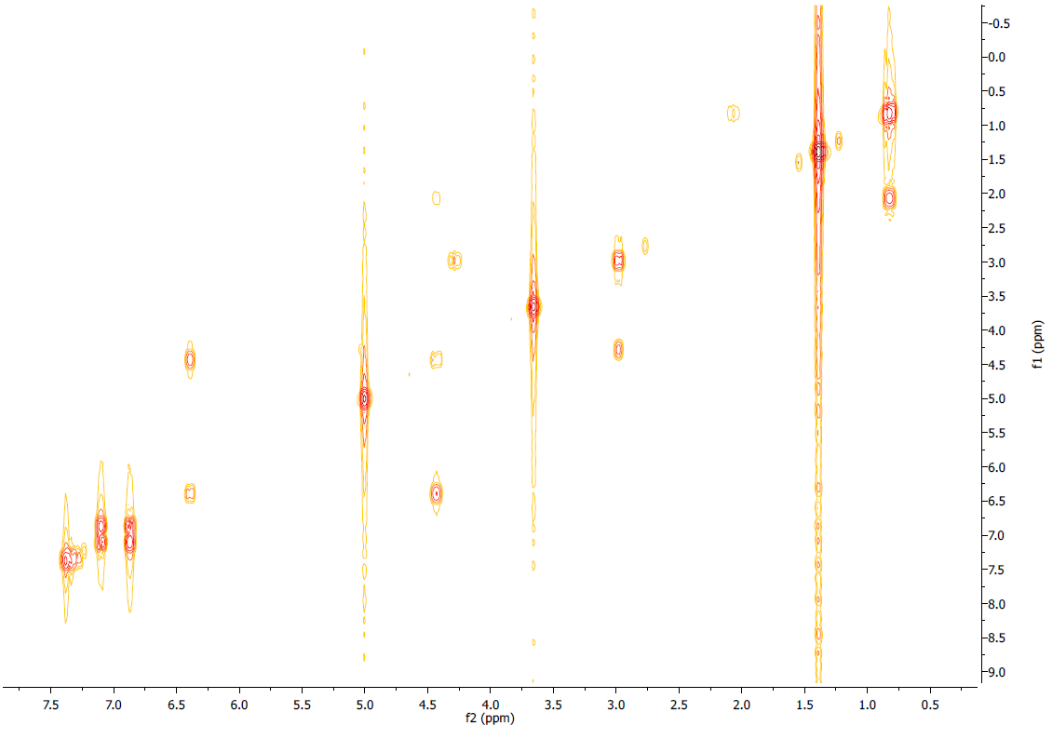

Boc-L-Tyr(Bn)-L-Val-OMe (^{13}C NMR)

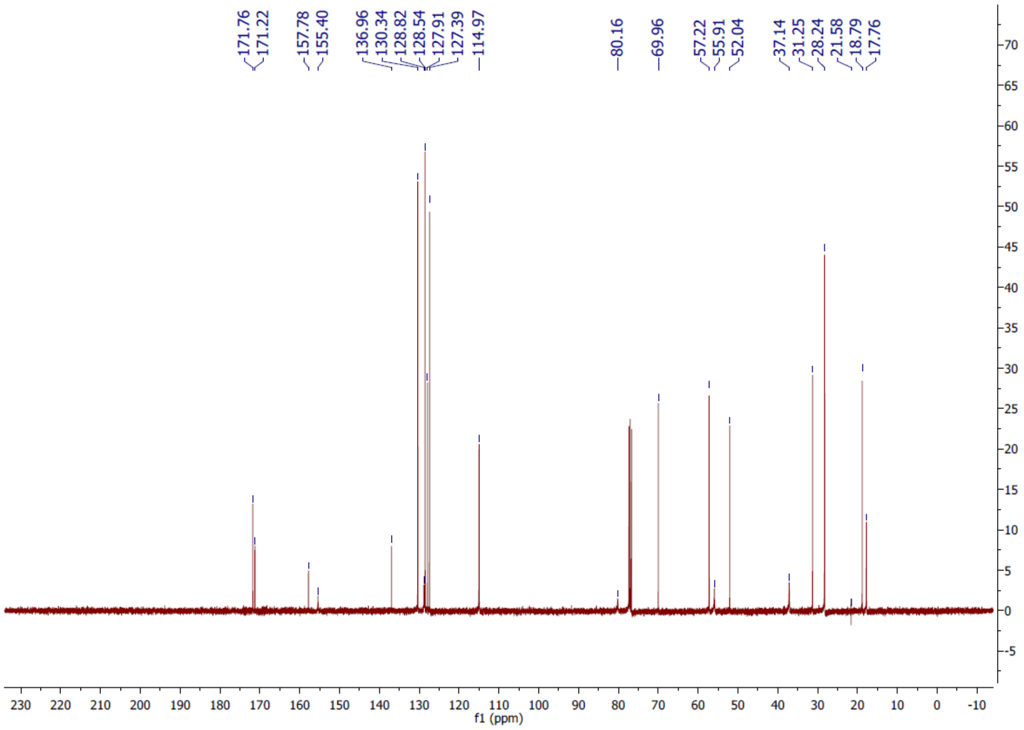

Boc-L-Tyr(Bn)-L-Val-OMe (HPLC-MS)

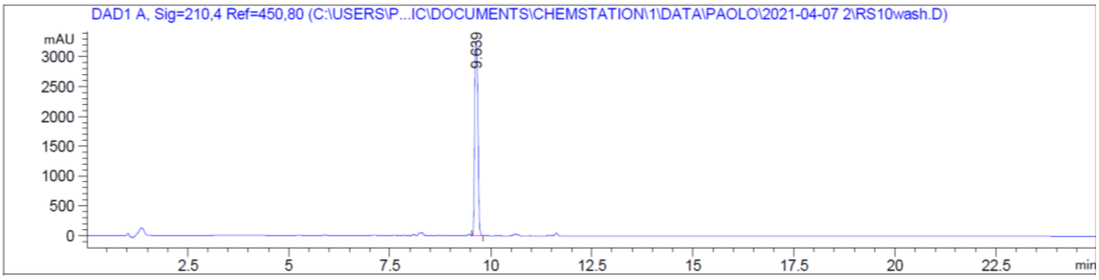

MS Spectrum

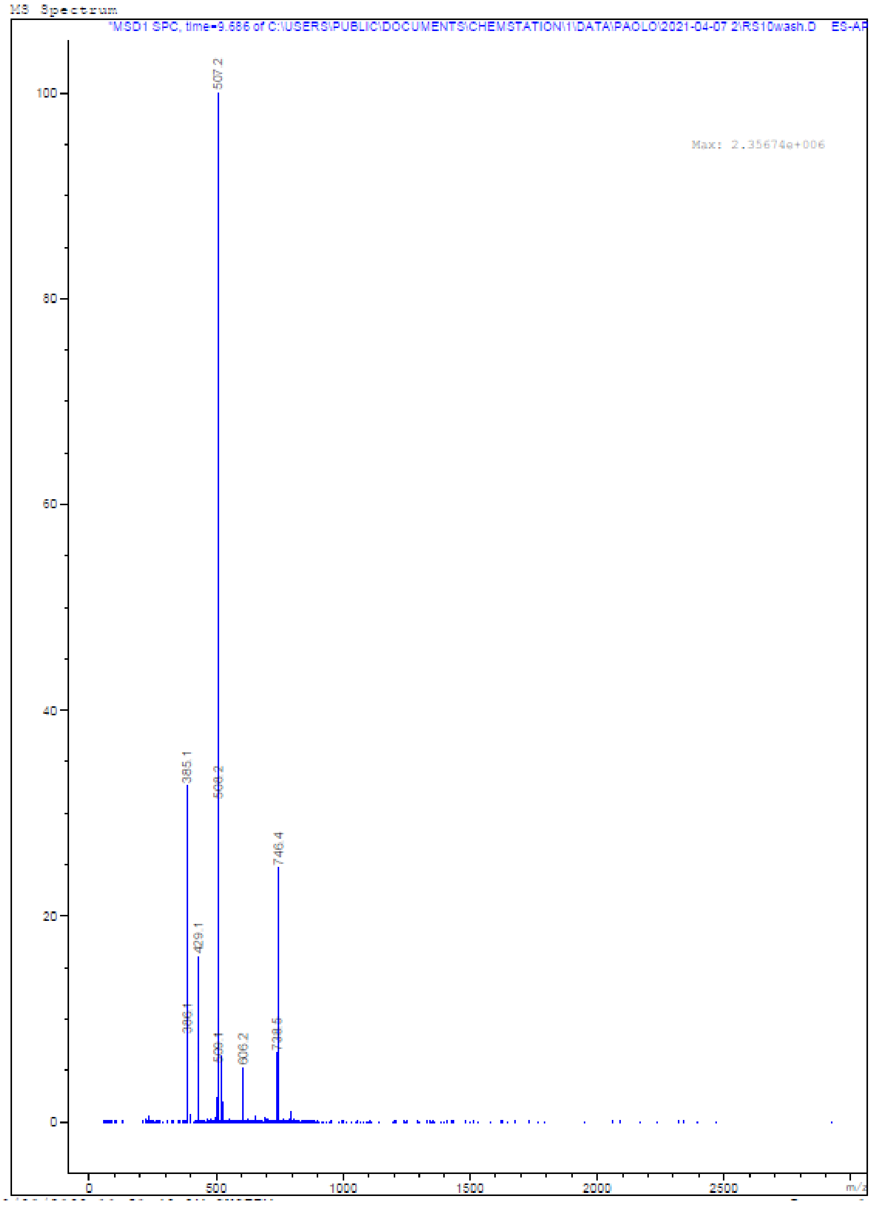

TFA-L-Val-L-Tyr(Bn)-L-Val-OMe (^{1}H NMR)

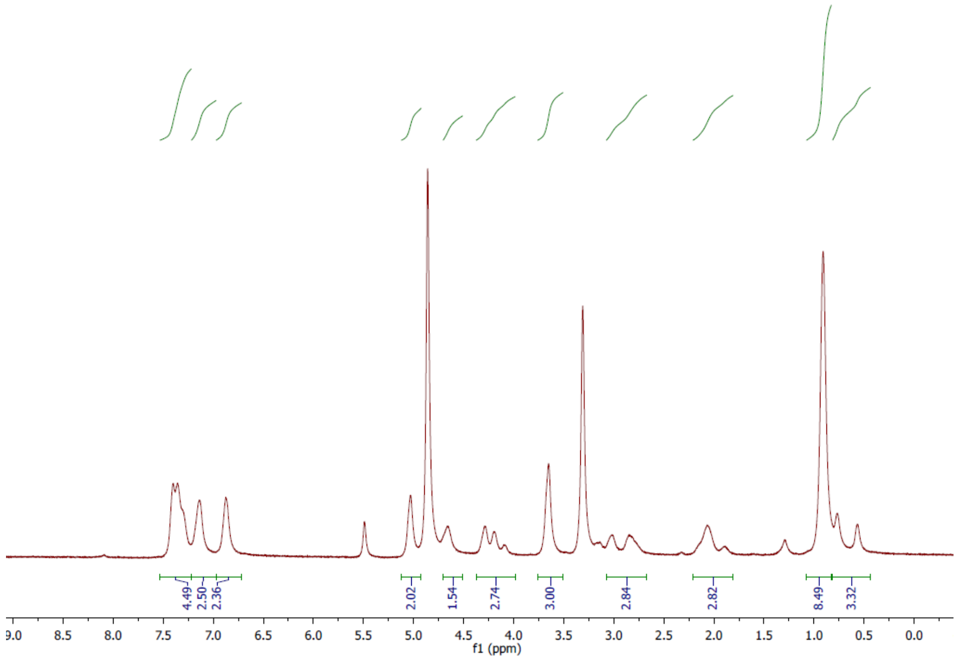

TFA-L-Val-L-Tyr(Bn)-L-Val-OMe (COSY)

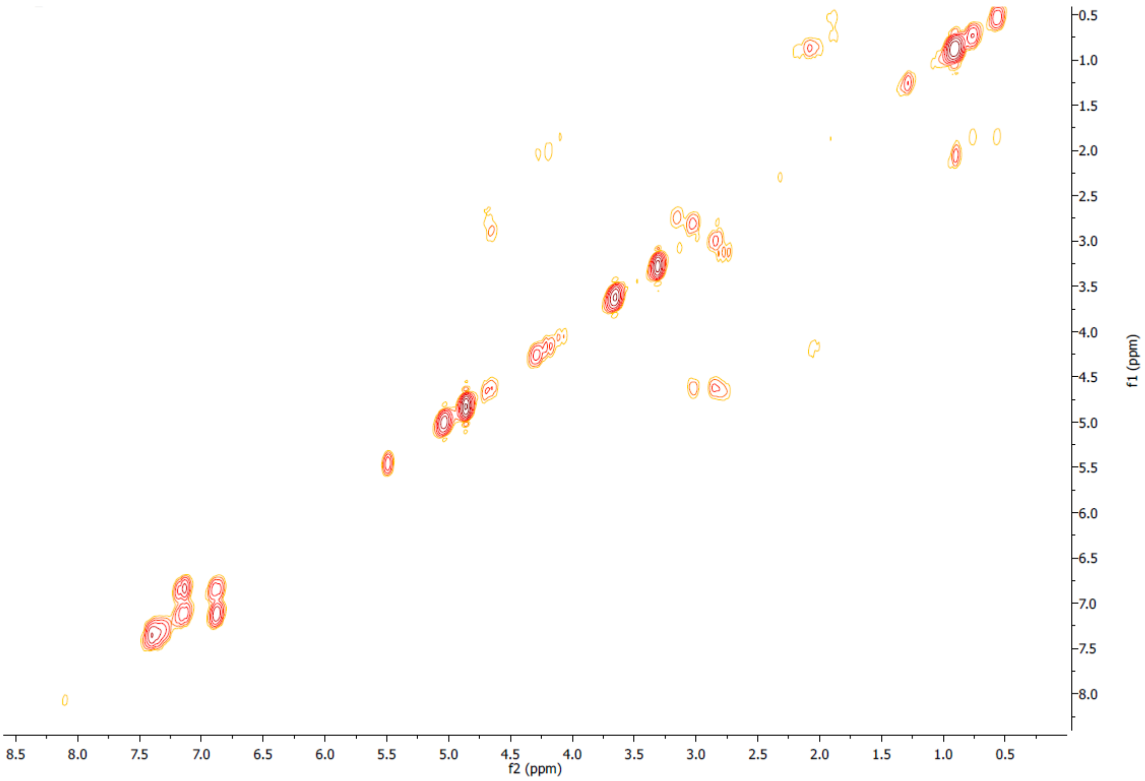

TFA-L-Val-L-Tyr(Bn)-L-Val-OMe (^{13}C NMR)

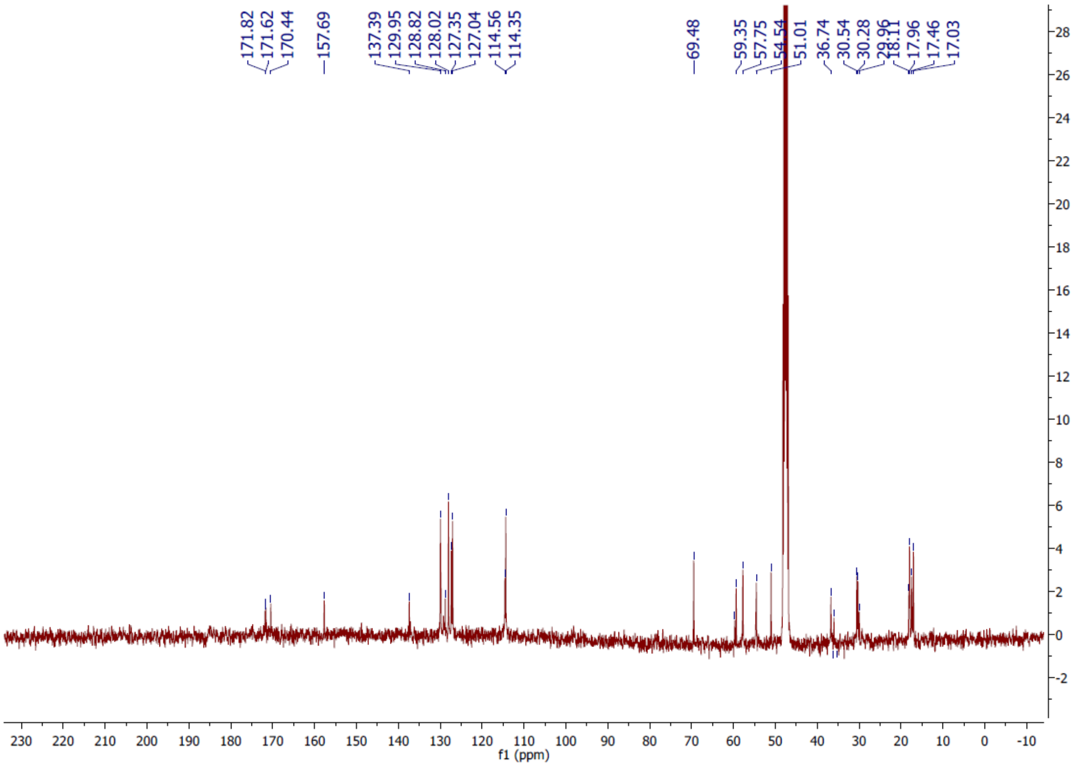

TFA-L-Val-L-Tyr(Bn)-L-Val-OMe (^{19}F NMR)

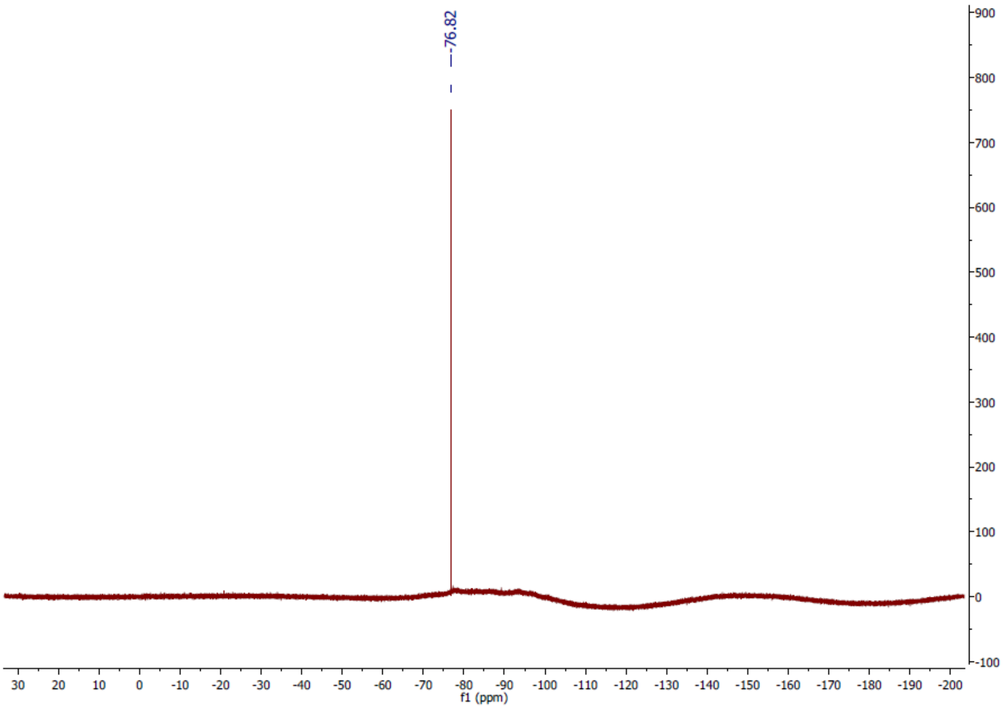

TFA-L-Val-L-Tyr(Bn)-L-Val-OMe (HPLC-MS)

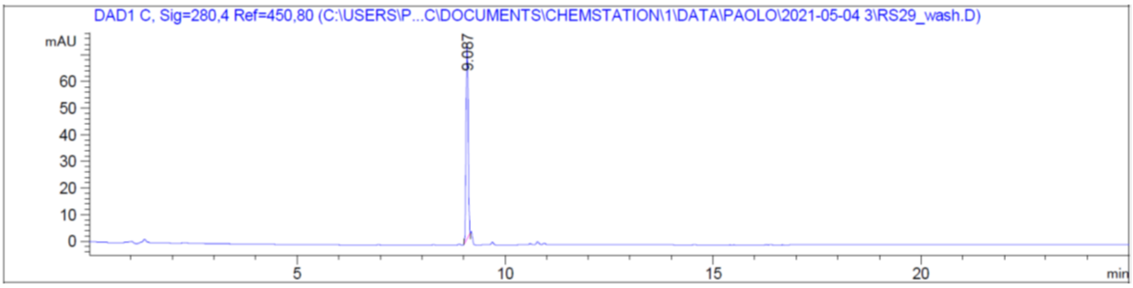

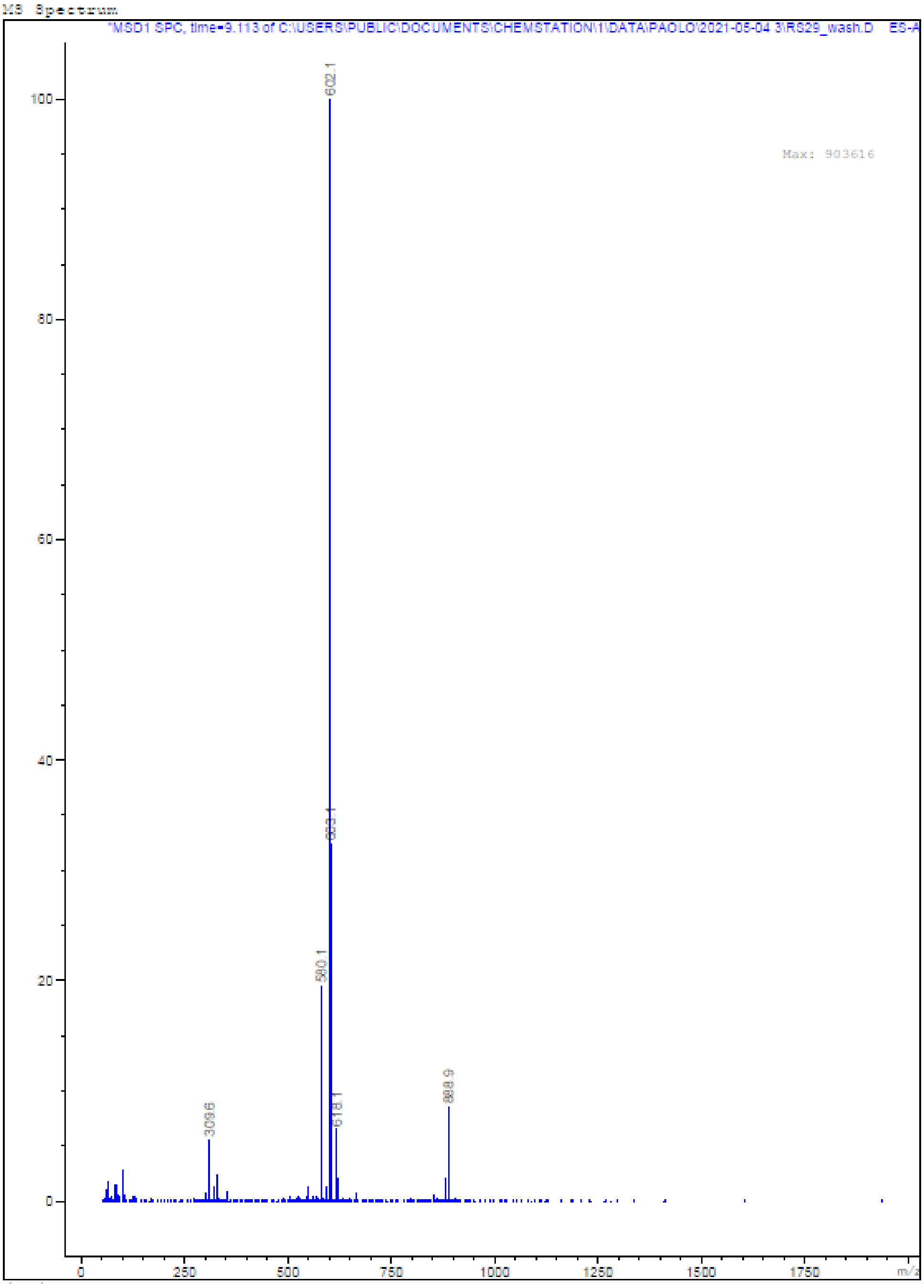

TFA-L-Val-L-Tyr-L-Val-OH (^{1}H NMR)

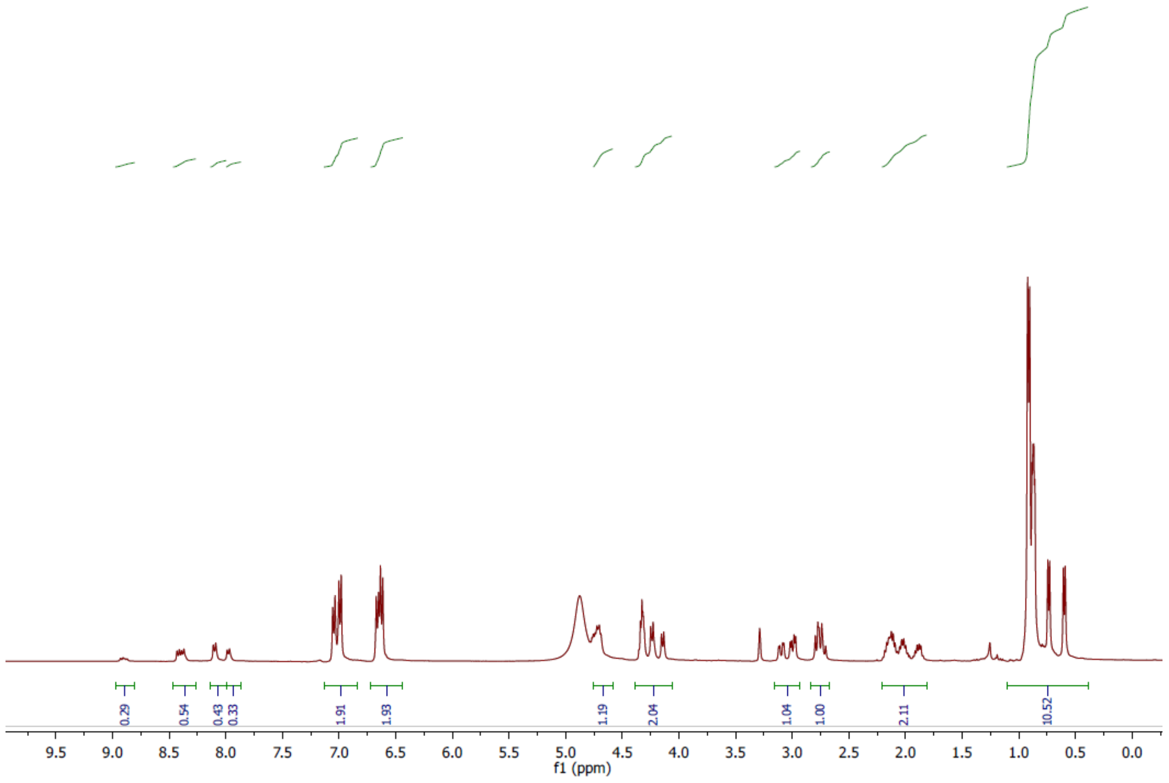

TFA-L-Val-L-Tyr-L-Val-OH (COSY)

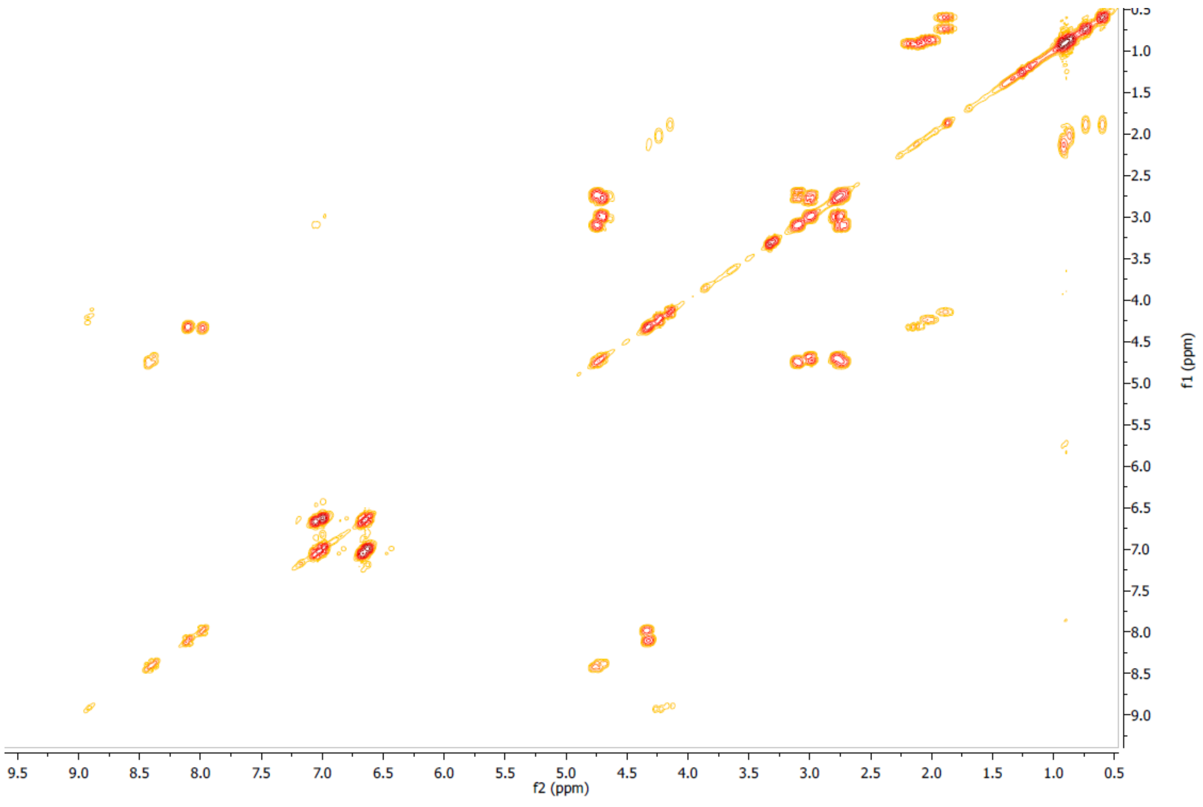

TFA-L-Val-L-Tyr-L-Val-OH (^{13}C NMR)

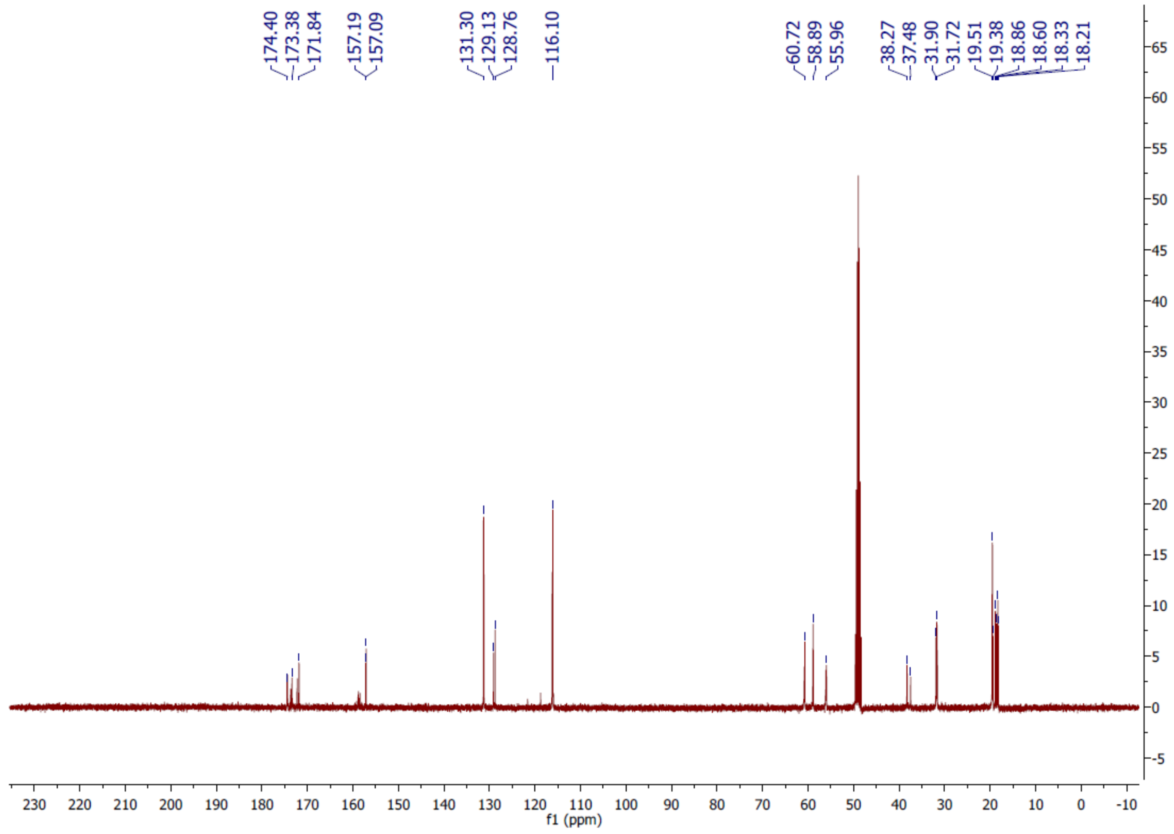

TFA-L-Val-L-Tyr-L-Val-OH (HPLC-MS)

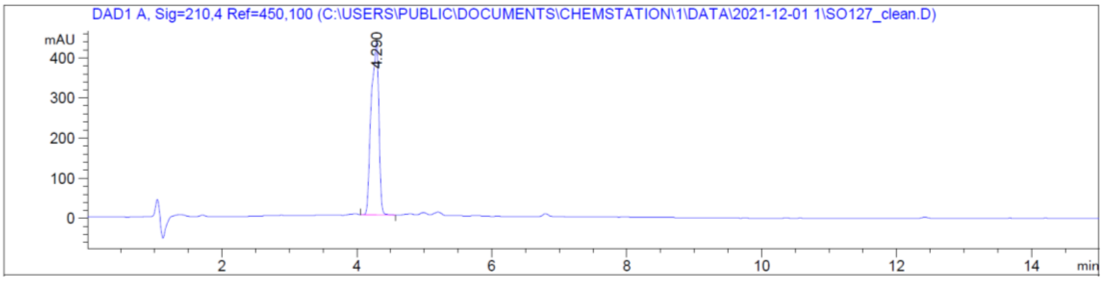

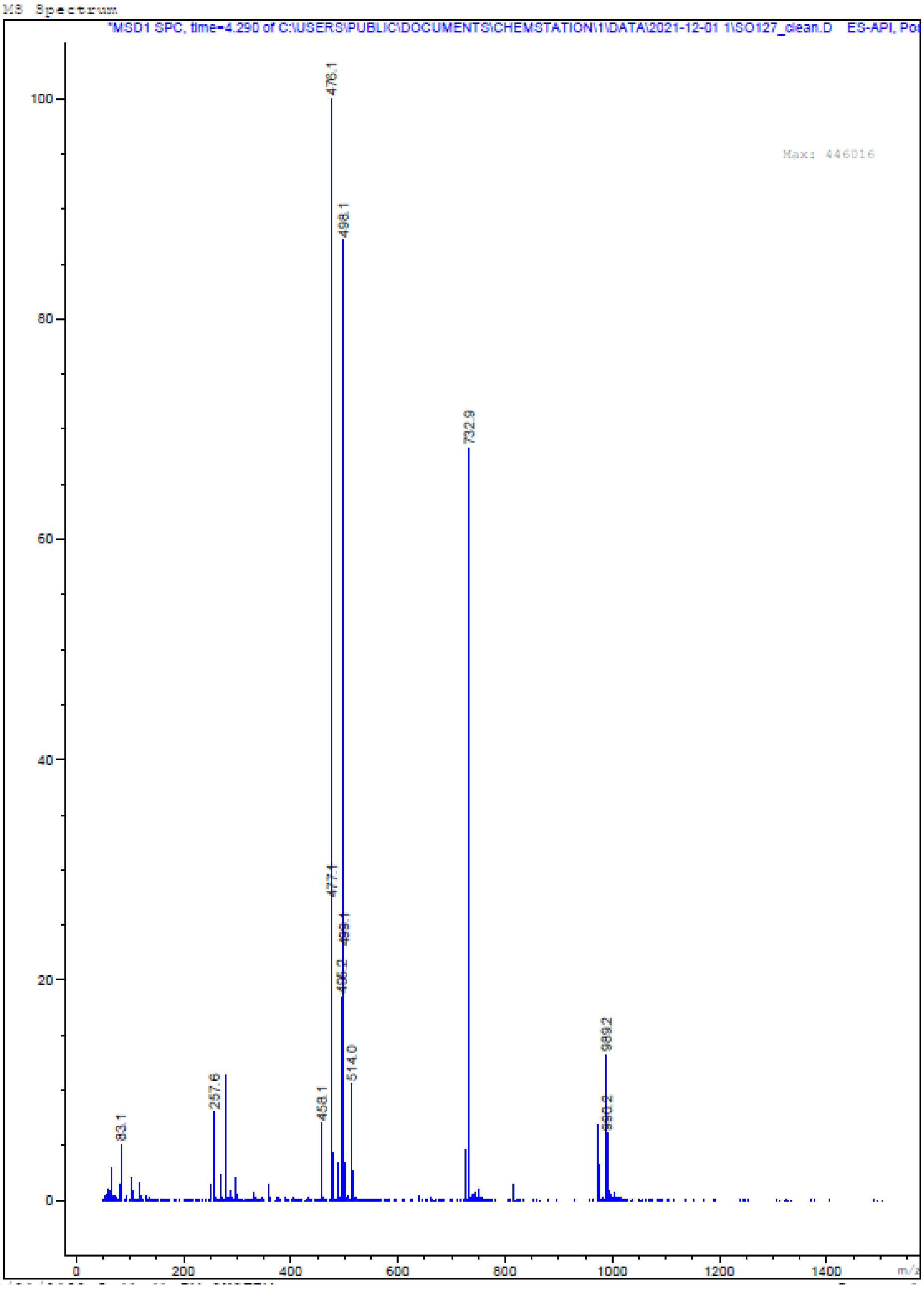

TFA-L-Val-L-Tyr-L-Val-OH (FTR-ATR)

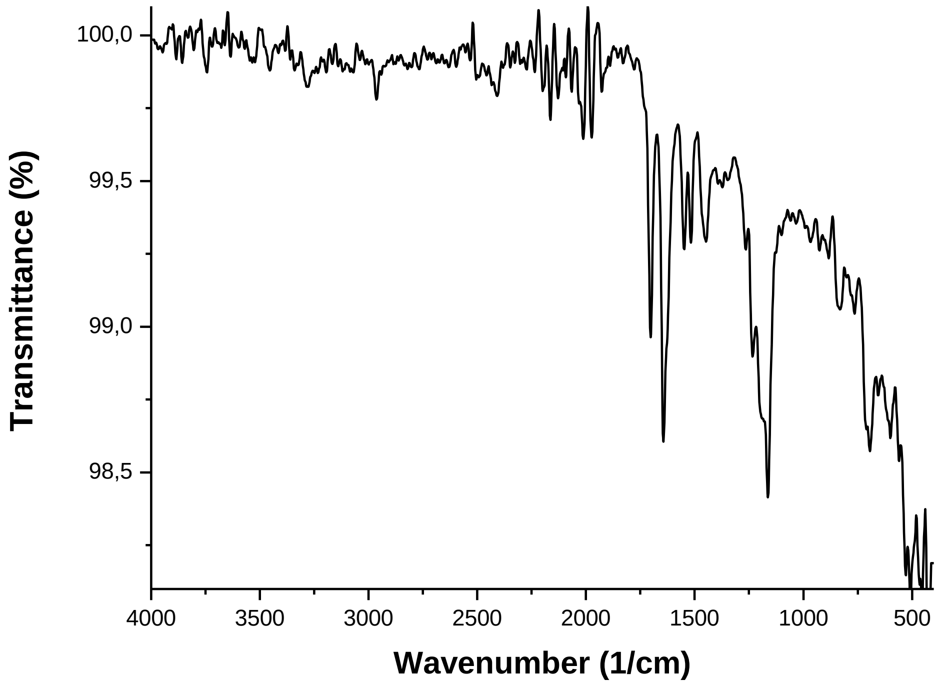

Boc-L-Lys(2Cl-Z)-OBn

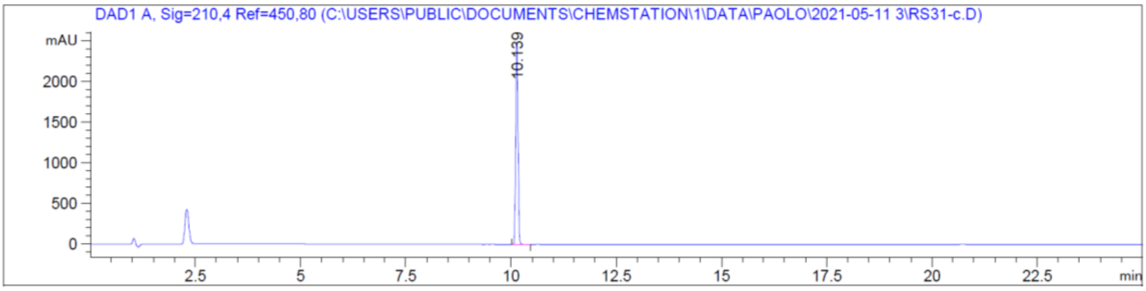

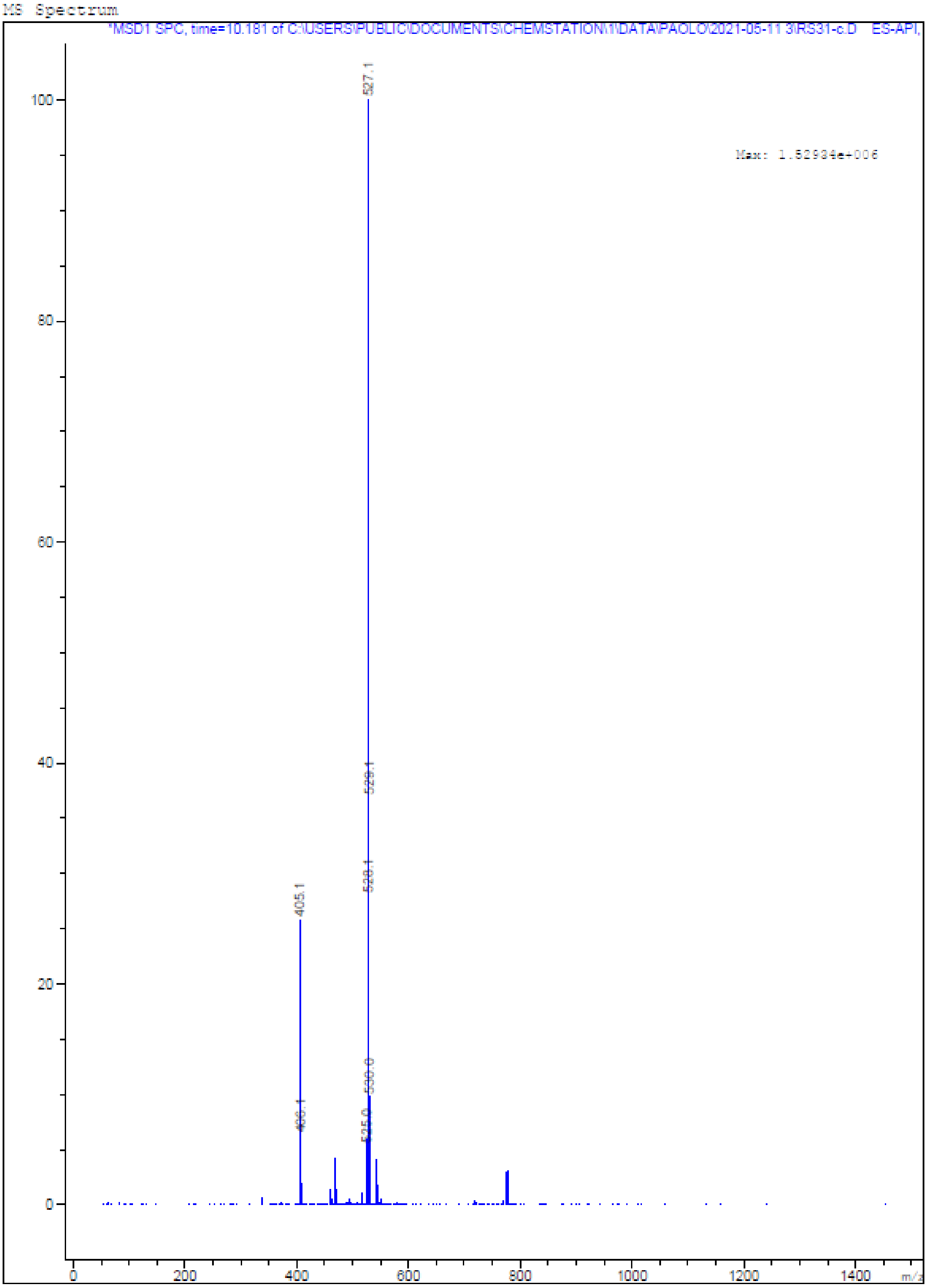

Boc-L-Lys(2Cl-Z)-L-Val-OH (^{1}H NMR)

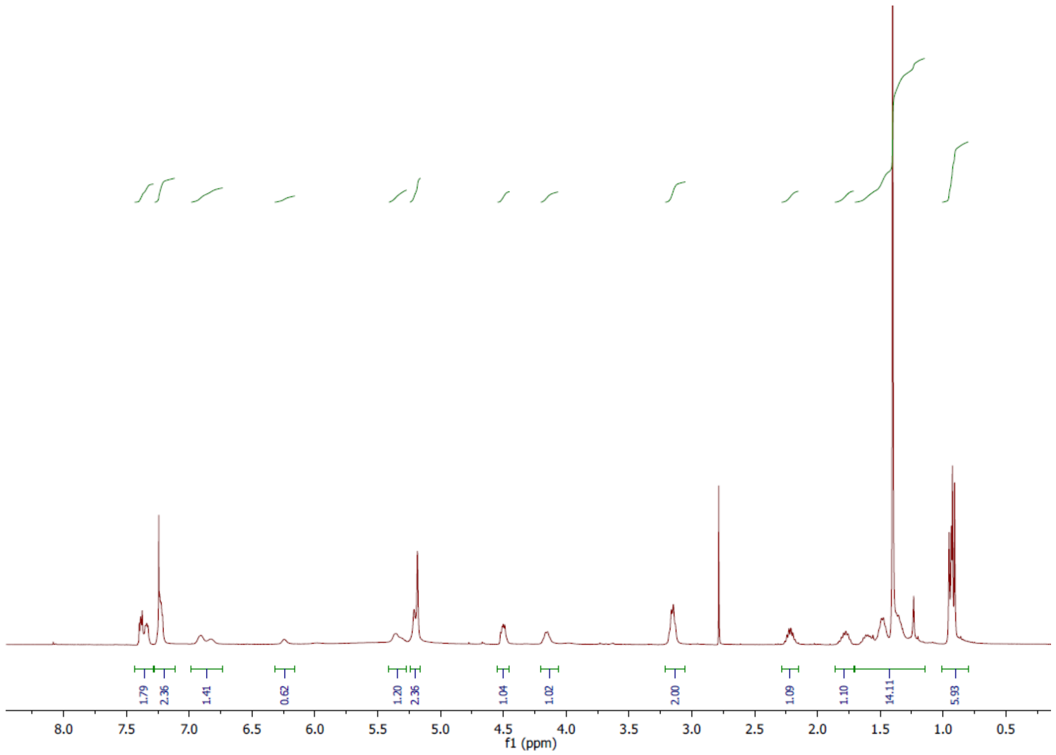

Boc-L-Lys(2Cl-Z)-L-Val-OH (^{13}C NMR)

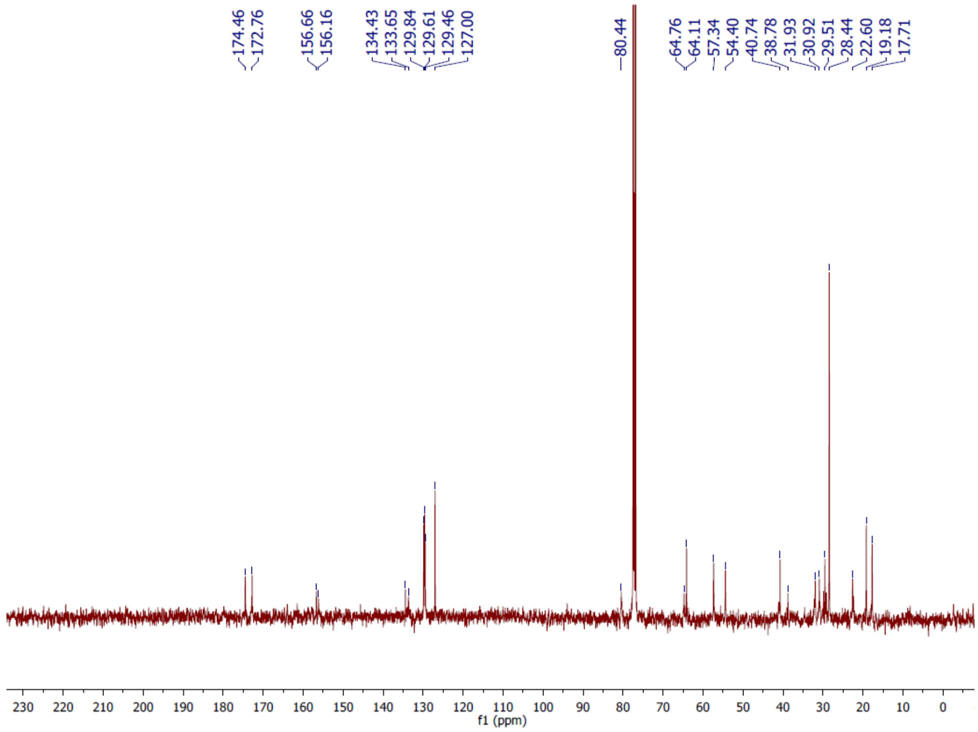

Boc-L-Lys(2Cl-Z)-L-Val-OH (HPLC-MS)

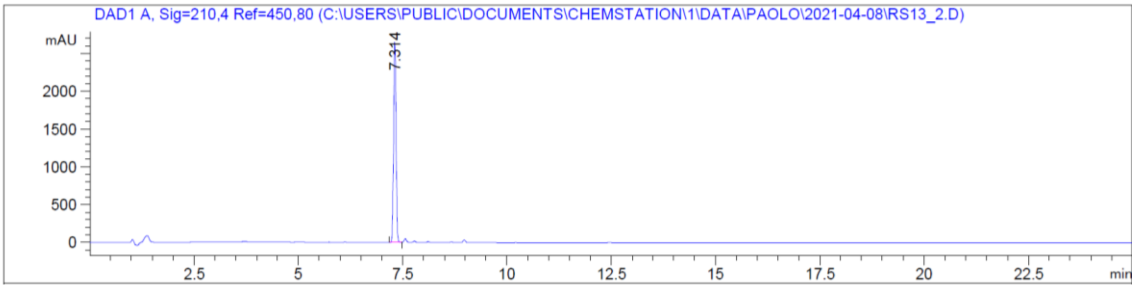

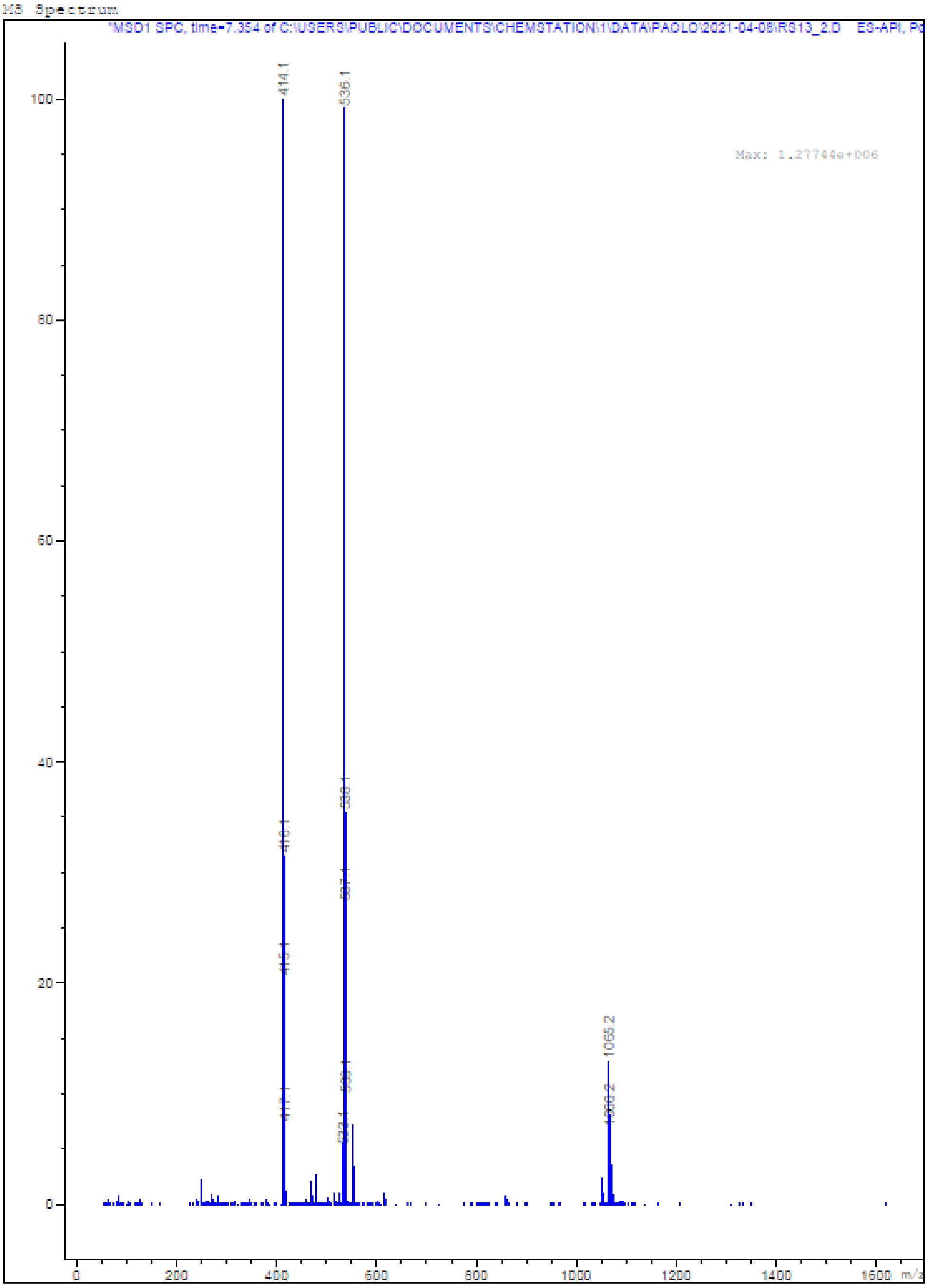

Boc-L-Lys(2Cl-Z)-OBn (HPLC-MS)

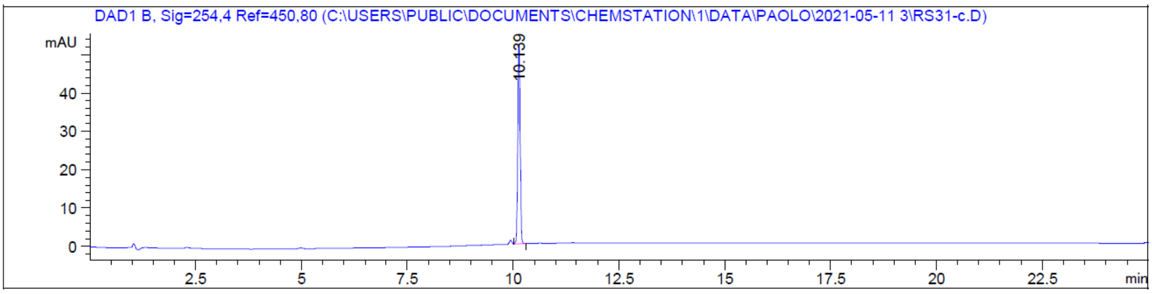

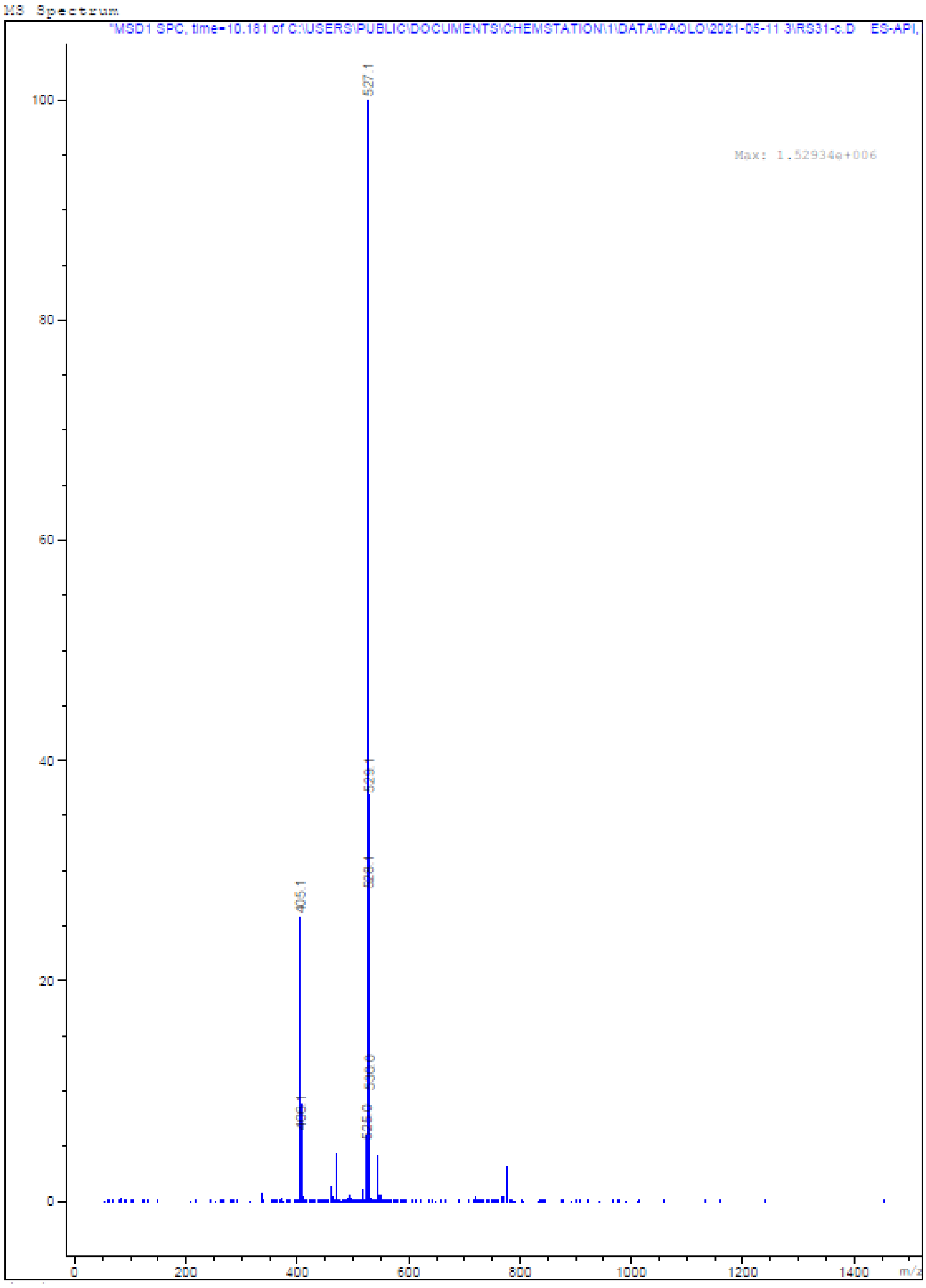

Boc-L-Lys(2Cl-Z)-L-Val-L-Lys(2Cl-Z)-OBn (^{1}H NMR)

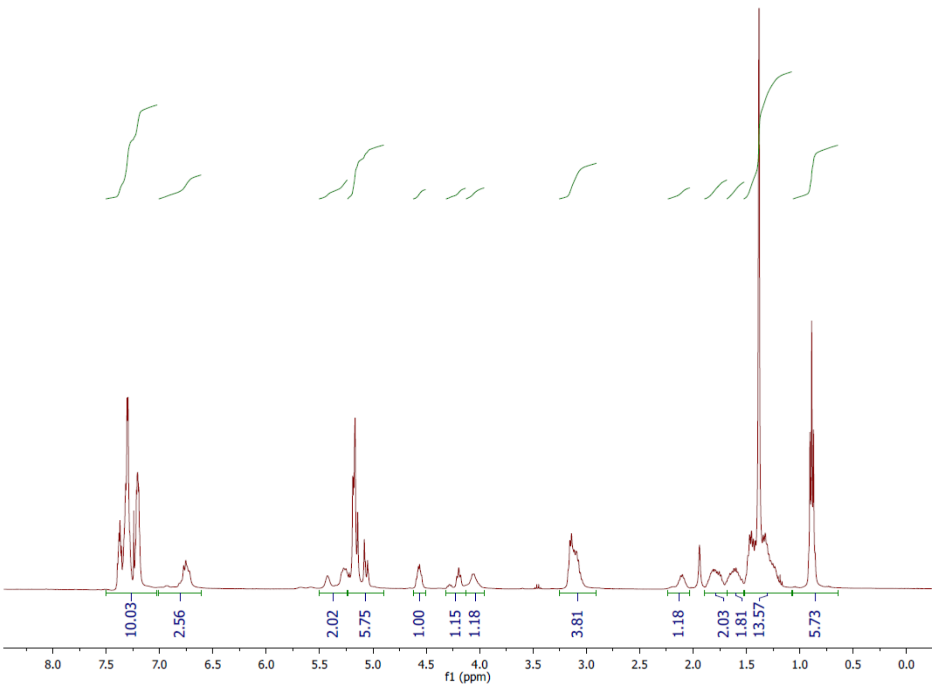

Boc-L-Lys(2Cl-Z)-L-Val-L-Lys(2Cl-Z)-OBn (COSY)

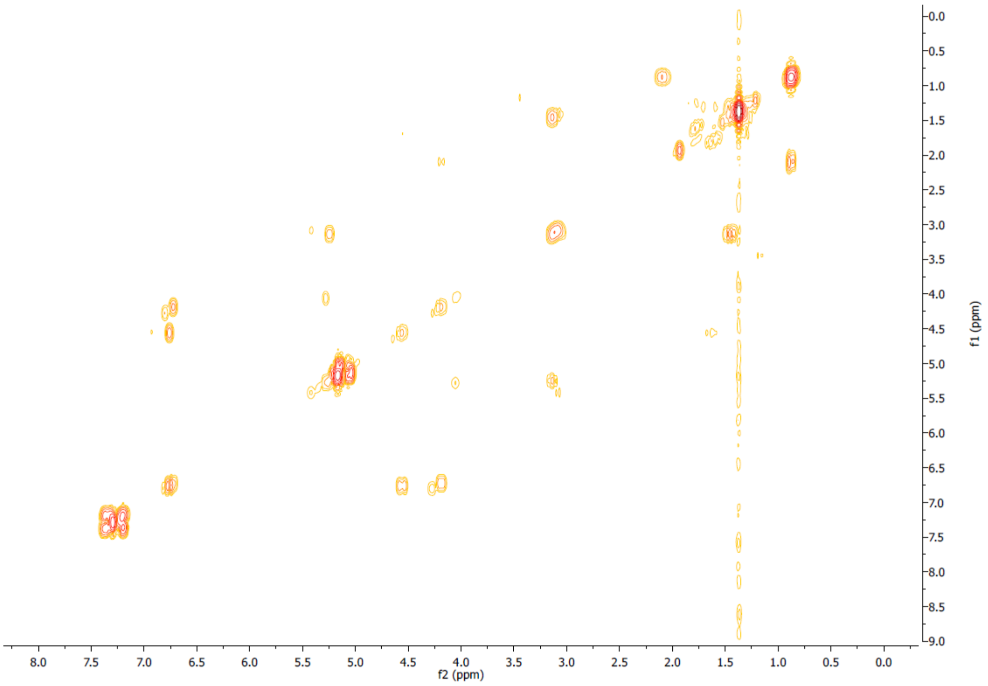

Boc-L-Lys(2Cl-Z)-L-Val-L-Lys(2Cl-Z)-OBn (^{13}C NMR)

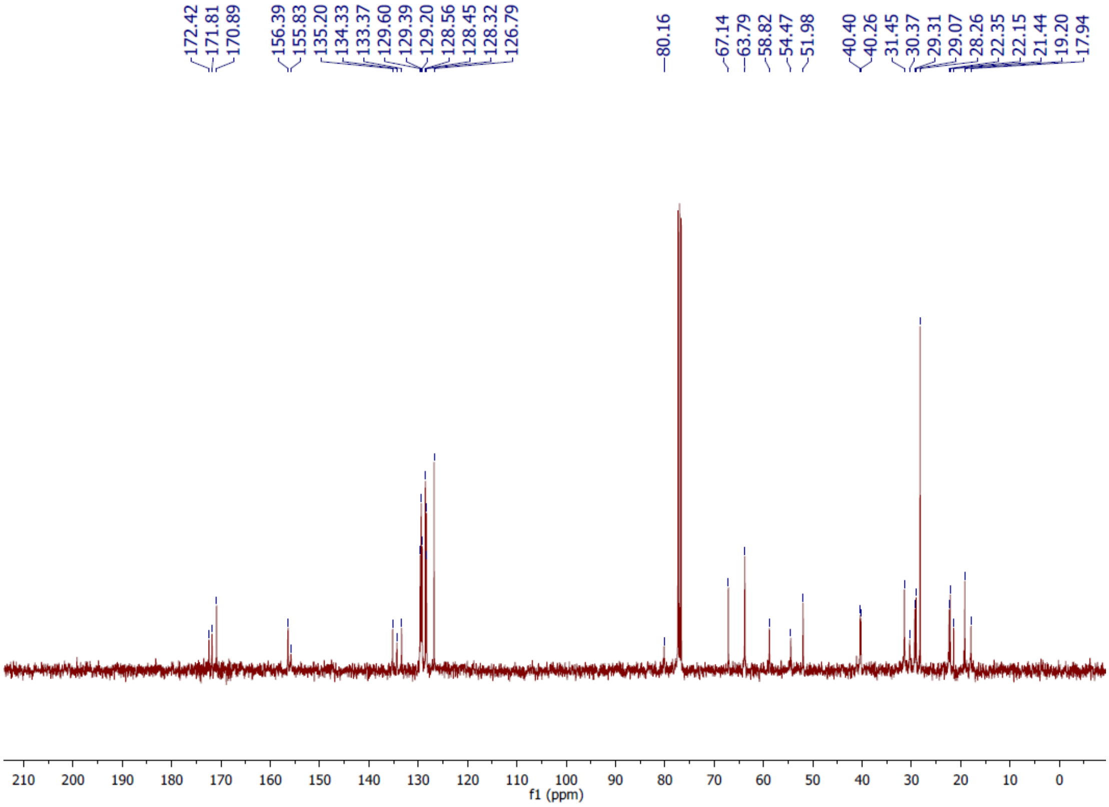

Boc-L-Lys(2Cl-Z)-L-Val-L-Lys(2Cl-Z)-OBn (HPLC-MS)

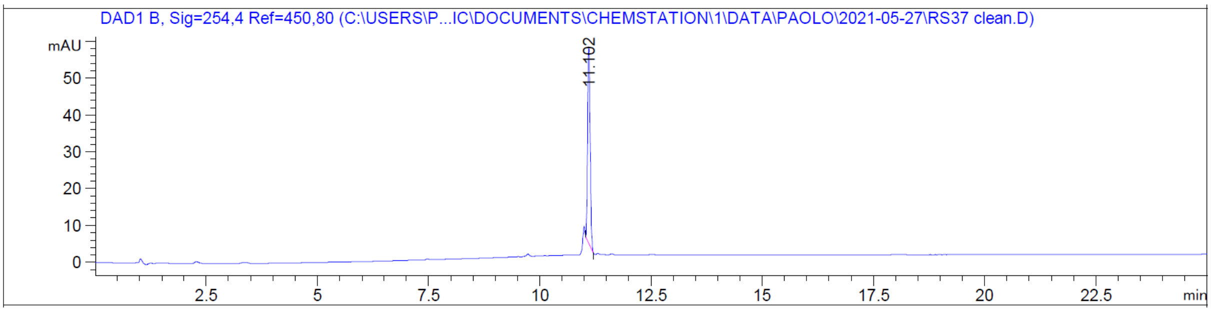

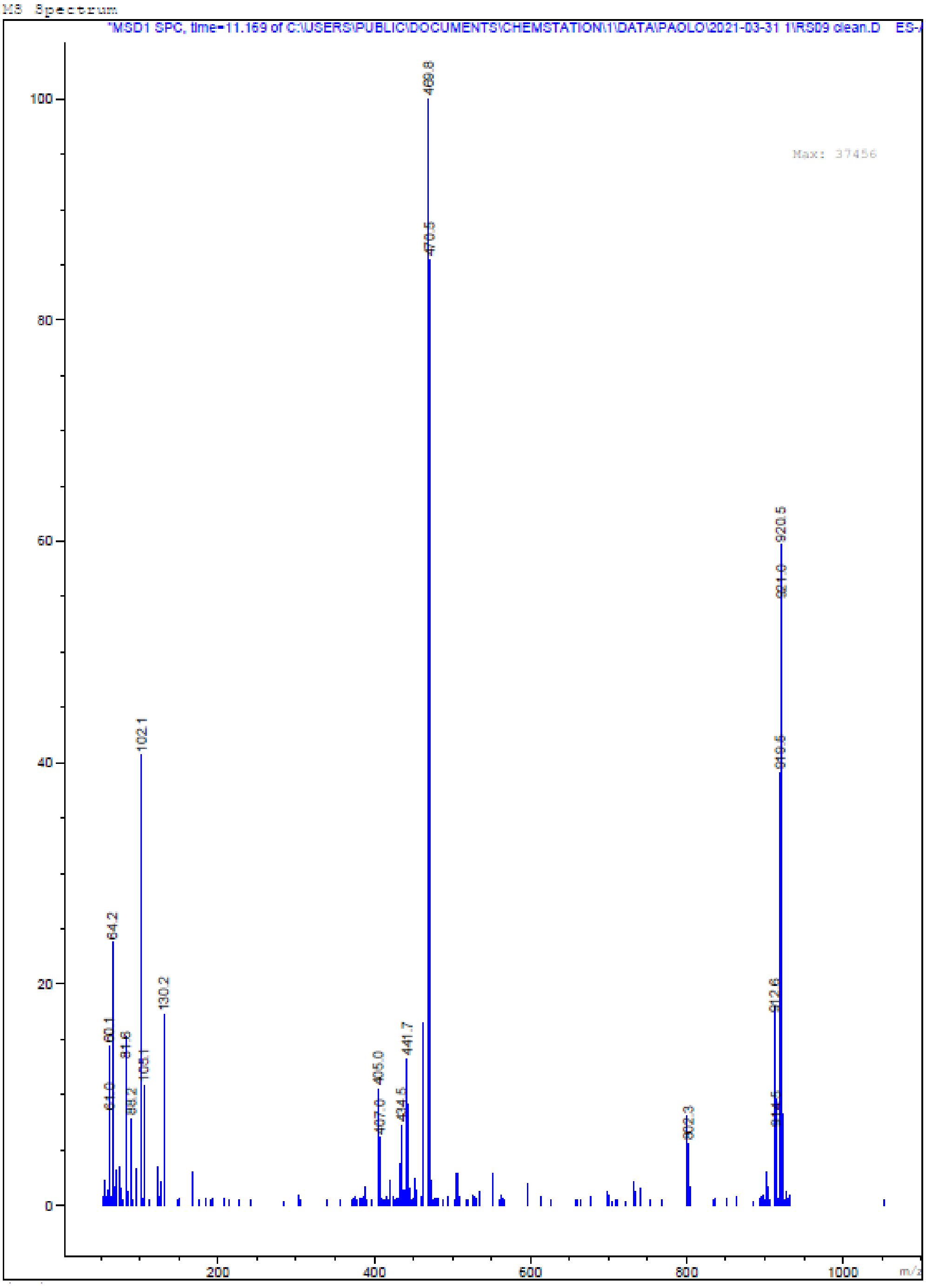

Pal-L-Lys(2Cl-Z)-L-Val-L-Lys(2Cl-Z)-OBn (^{1}H NMR)

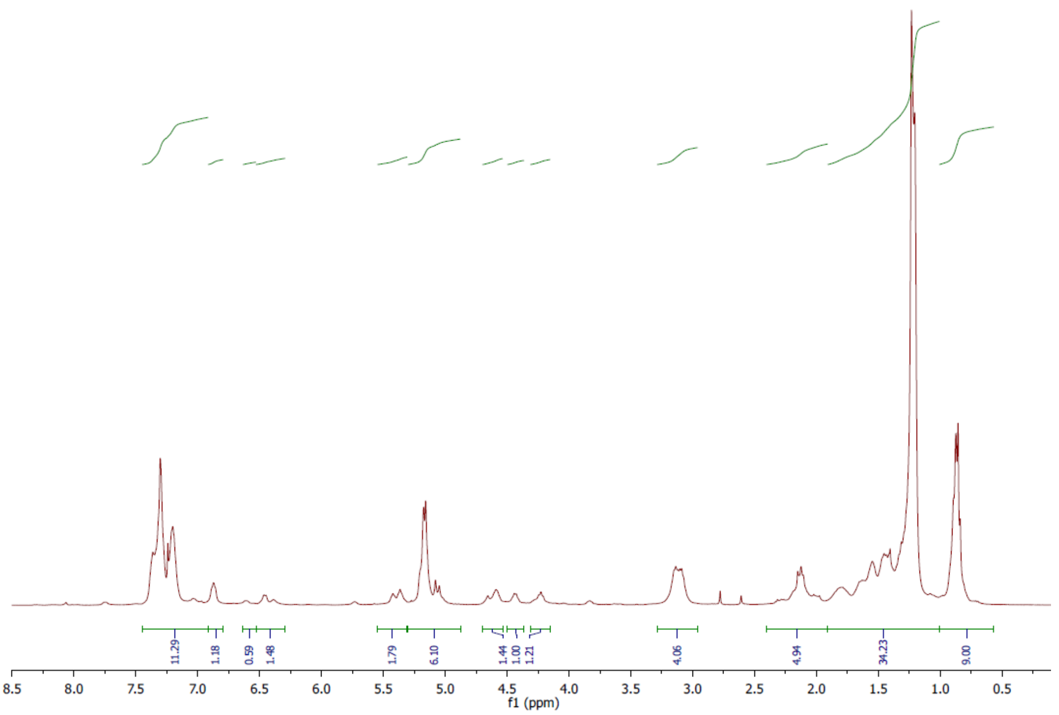

Pal-L-Lys(2Cl-Z)-L-Val-L-Lys(2Cl-Z)-OBn (COSY)

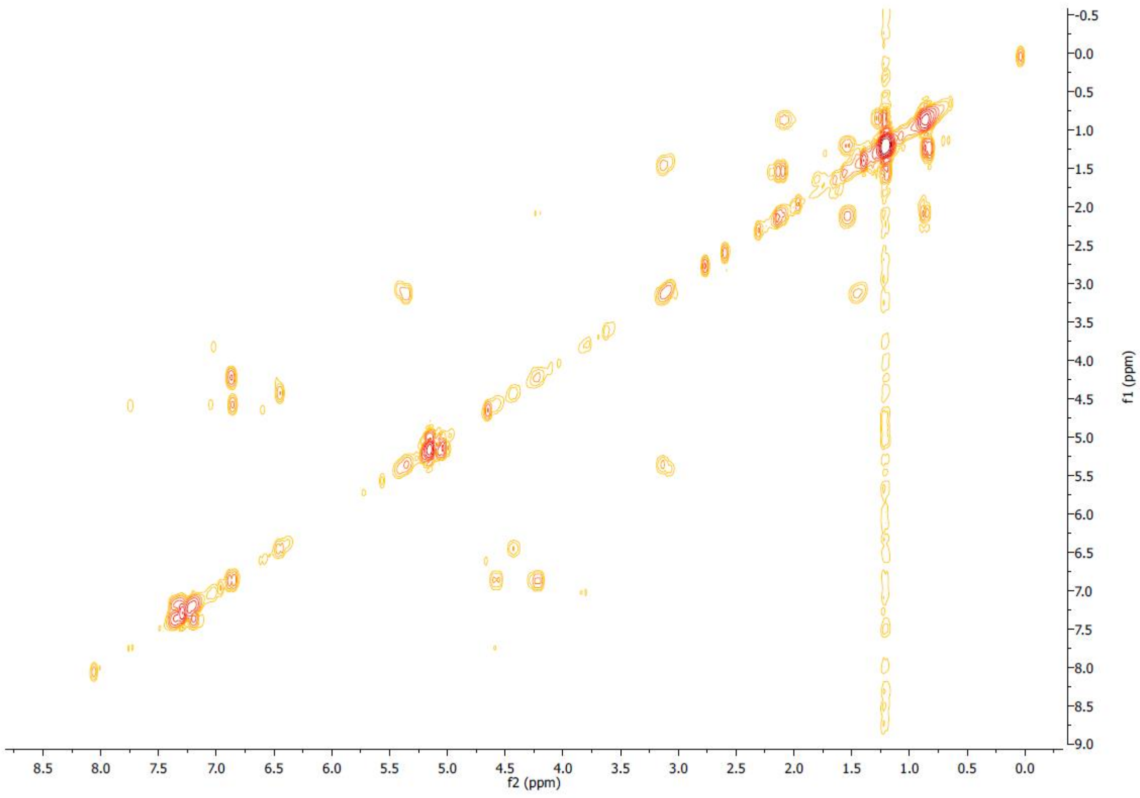

Pal-L-Lys(2Cl-Z)-L-Val-L-Lys(2Cl-Z)-OBn (^{13}C NMR)

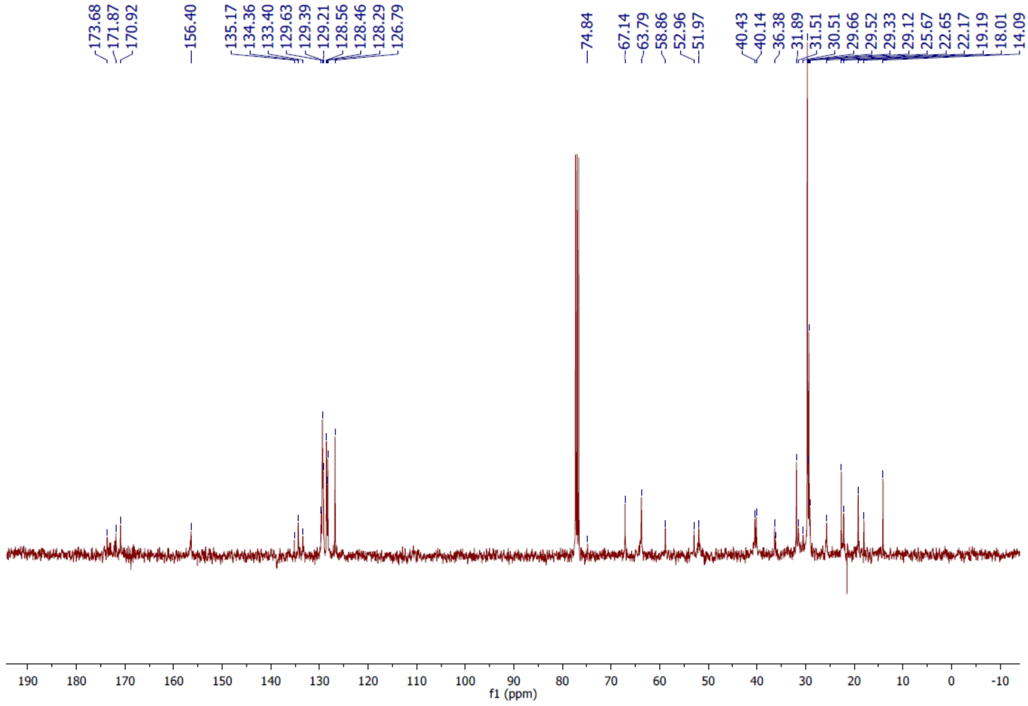

Pal-L-Lys-L-Val-L-Lys-OH (^{1}H NMR)

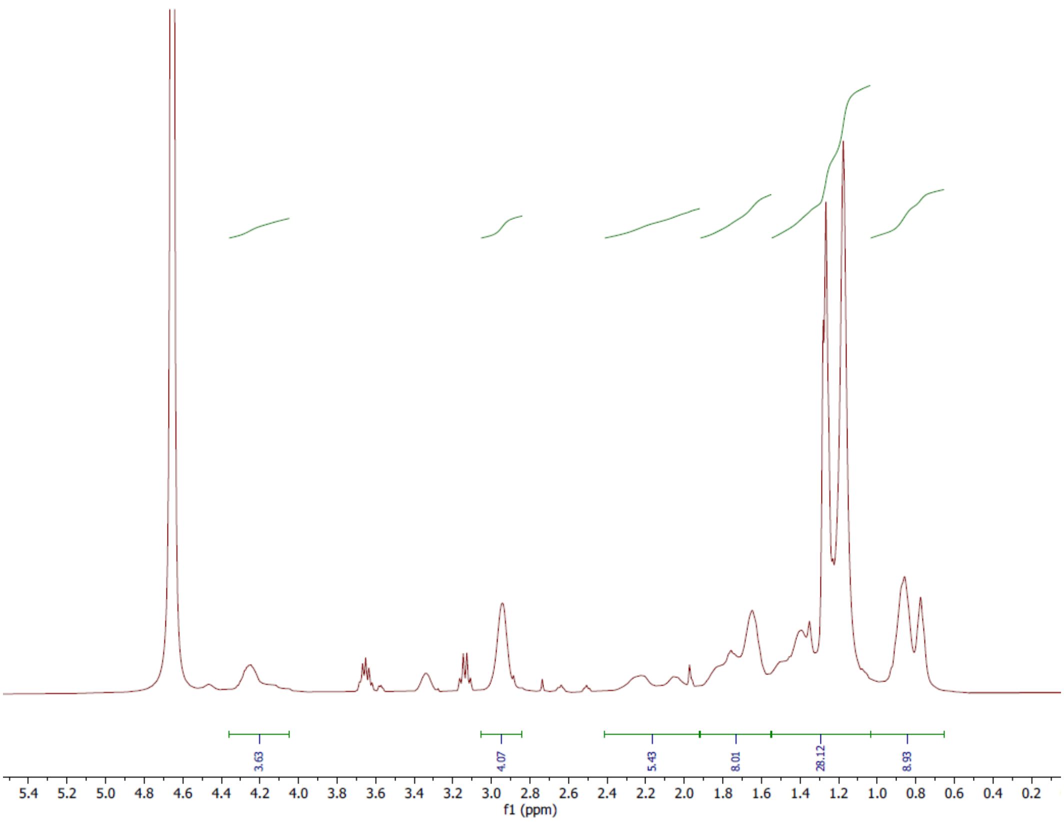

Pal-L-Lys-L-Val-L-Lys-OH (^{13}C NMR)

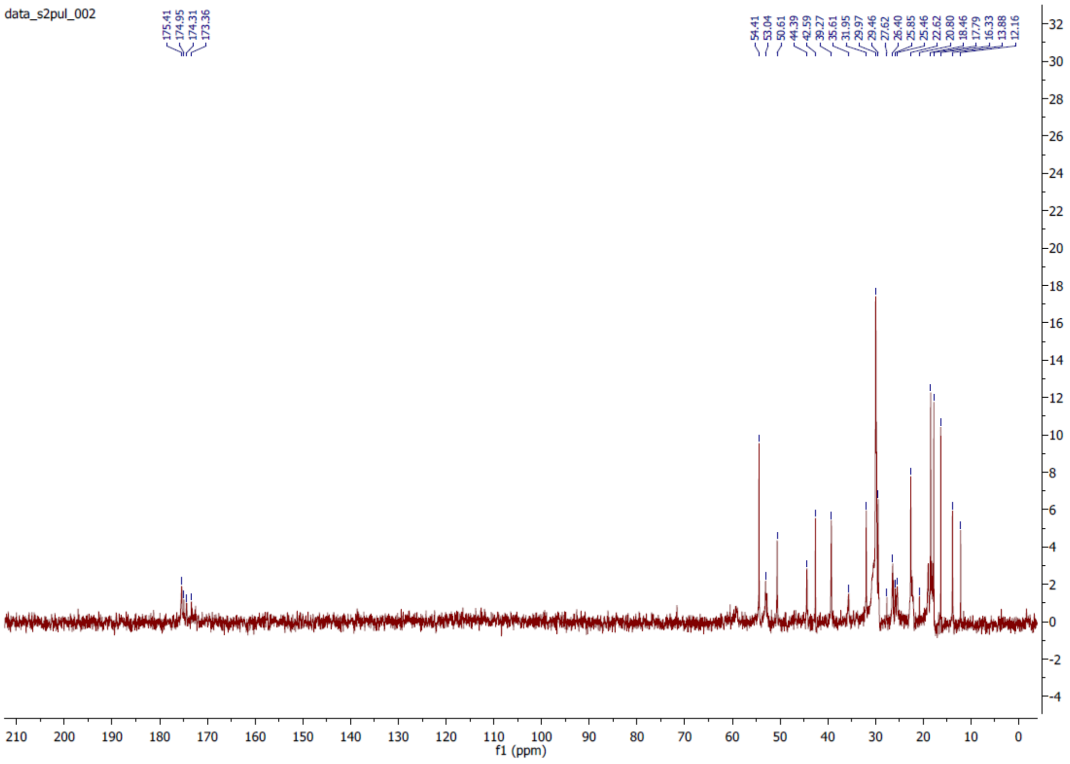

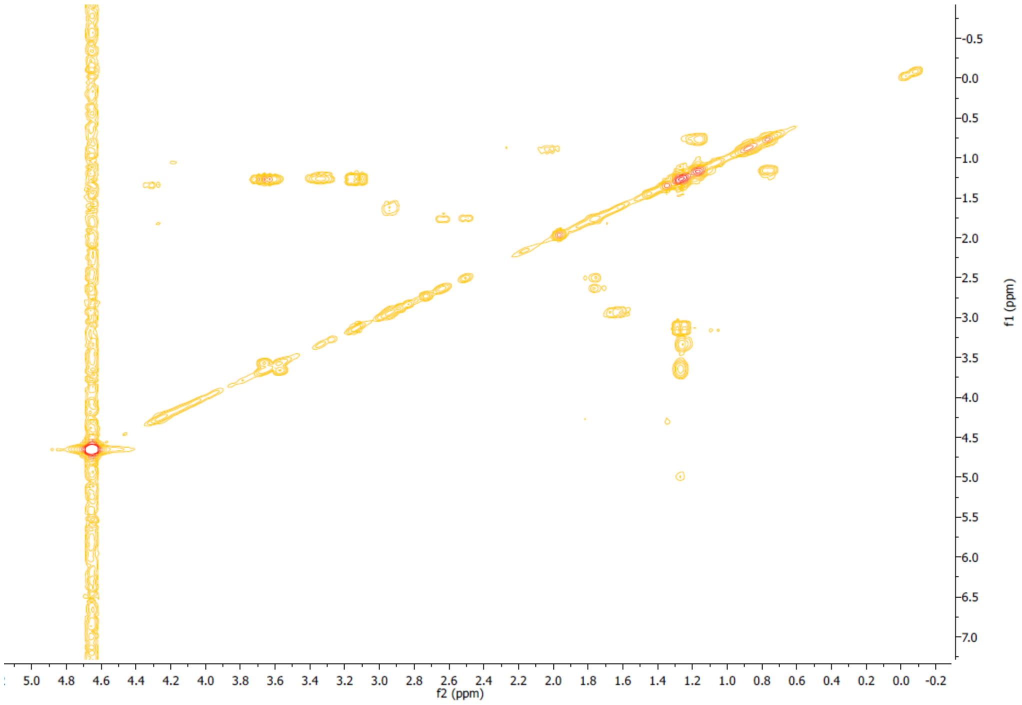
5.0 4.8 4.6 4.4 4.2 4.0 3.8 3.6 3.4 3.2 3.0 2.8 2.6 2.4 2.2 2.0 1.8 1.6 1.4 1.2 1.0 0.8 0.6 0.4 0.2 0.0 -0.2
f2 (ppm)
-0.5 0.0 0.5 1.0 1.5 2.0 2.5 3.0 3.5 4.0 4.5 5.0 5.5 6.0 6.5 7.0
f1 (ppm)

Pal-L-Lys-L-Val-L-Lys-OH (HPLC-MS)

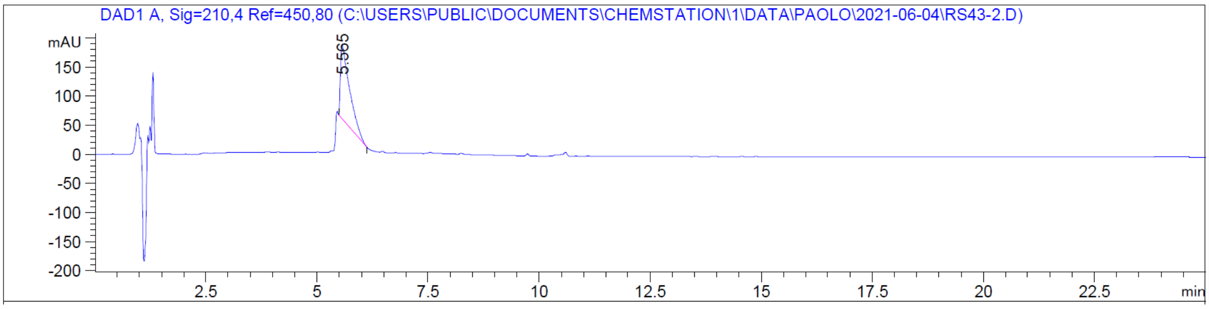

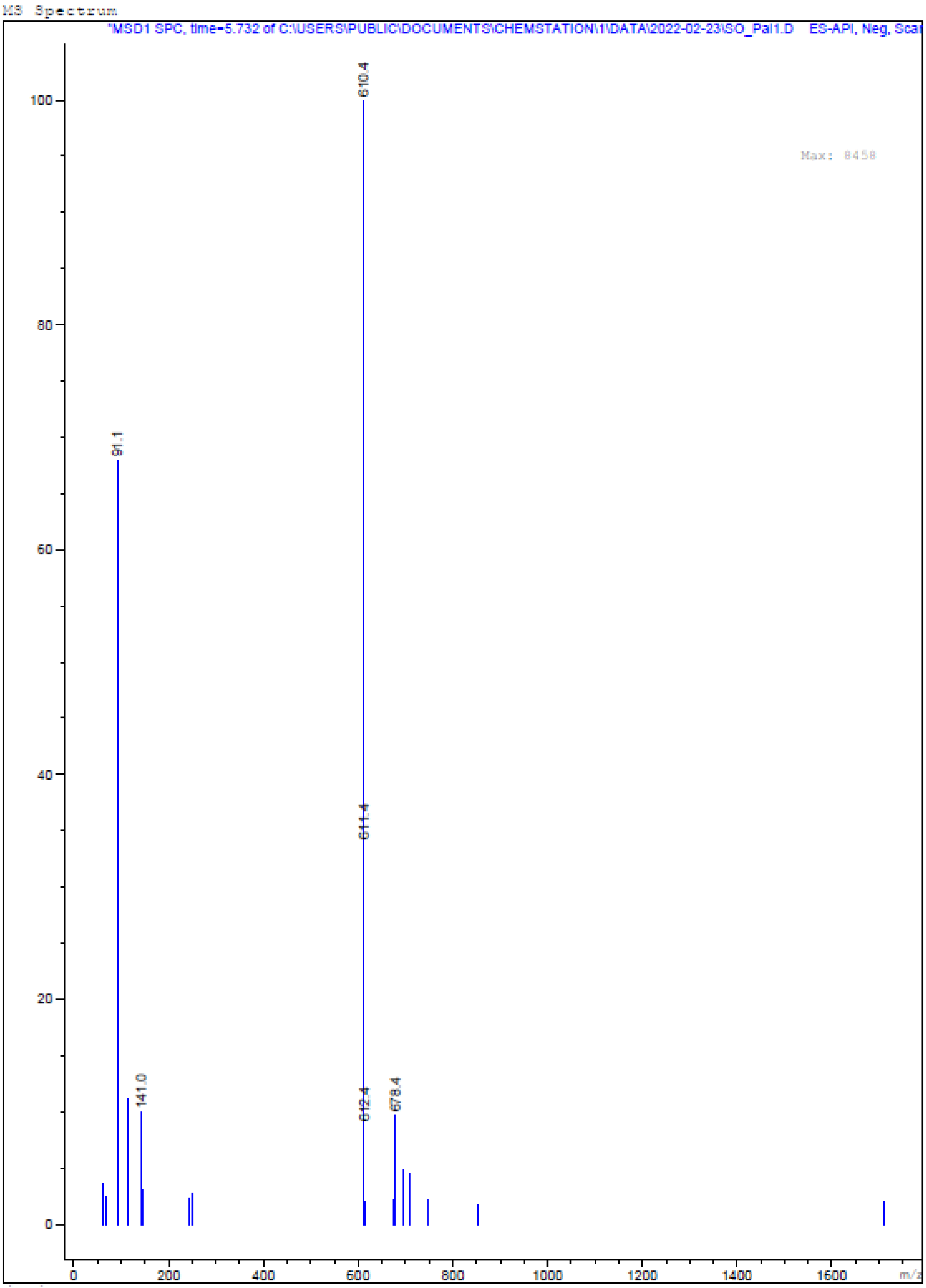

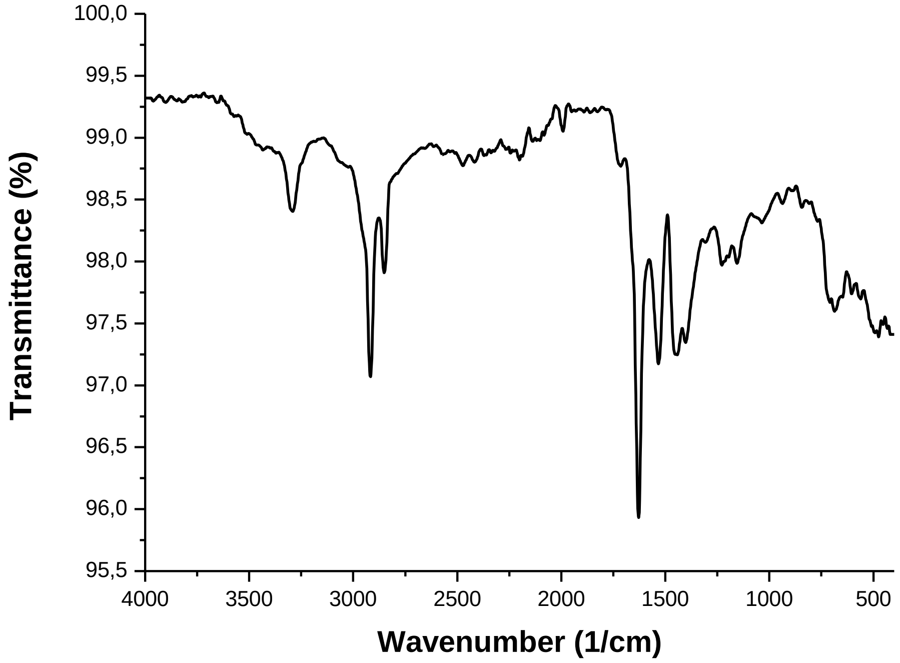
100,0
99,5
99,0
98,5
98,0
97,5
97,0
96,5
96,0
95,5
Transmittance (%)
4000
3500
3000
2500
2000
1500
1000
500
Wavenumber (1/cm)
